# Supplementary material for: Agenda-setting in the clinical encounter: A systematic review protocol
Source: PLoS One. 2024 Oct 24;19(10):e0312613. doi: 10.1371/journal.pone.0312613 (PMC11500969; doi:10.1371/journal.pone.0312613)
Supplement: S2 File — (DOCX) [file pone.0312613.s002.docx]

## Supporting Information 2. Piloted search strategy

### Keywords

***Table 1.*** *Search strategy themes and keywords*

| **Theme** | **Keywords** |
| --- | --- |
| Clinical context | Patient, clinician, clinical, clinical visit, clinical encounter |
| Agenda-setting | Agenda setting, agenda mapping, agenda elicitation, agenda solicitation, topic setting, topic mapping, topic elicitation, topic solicitation, concern setting, concern mapping, concern elicitation, concern solicitation, priority setting, priority mapping, priority elicitation, priority solicitation, patient agenda, patient topics, patient concerns, patient priorities, opening statement, visit opening, visit structure, encounter opening, encounter structure |
| Interventions | Approach, brochure, checklist, coaching, conversation analysis, conversation approach, dashboard, education, electronic health record, electronic medical record, form, framework, guide, handout, information, instrument, intervention, issue card, material, model, pamphlet, portal, prompt, protocol, question asking, questionnaire, seminar, session, strategy, survey, technique, template, tool, training, webinar, workflow, worksheet, workshop |

### Database searches

**Database**: APA PsycInfo

**Dates covered**: 1806 to present

**Date searched**: October 2023

**Limits**: None used.

**Search terms / results**: We searched abstracts in PsycInfo with the queries below. The search returned 209 results.

***Table 2.*** *APA PsycInfo search queries and results*

| **Search** | **Query** | **Results** |
| --- | --- | --- |
| 1 | AB "agenda setting" OR AB "set agenda*" OR AB "setting agenda*" | 804 |
| 2 | AB "agenda mapping" OR AB "map agenda*" OR AB "mapping agenda*" | 8 |
| 3 | AB "agenda elicit*" OR AB "elicit agenda*" OR AB "eliciting agenda*" OR AB "elicited agenda*" | 2 |
| 4 | AB "agenda solicit*" OR AB "solicit agenda*" OR AB "soliciting agenda*" OR AB "solicited agenda*" | 1 |
| 5 | AB "topic setting" OR AB "set topic*" OR AB "setting topic*" | 52 |
| 6 | AB "topic mapping" OR AB "map topic*" OR AB "mapping topic*" | 8 |
| 7 | AB "topic elicit*" OR AB "elicit topic*" OR AB "eliciting topic*" OR AB "elicited topic*" | 17 |
| 8 | AB "topic solicit*" OR AB "solicit topic*" OR AB "soliciting topic*" OR AB "solicited topic*" | 4 |
| 9 | AB "concern setting" OR AB "set concern*" OR AB "setting concern*" | 72 |
| 10 | AB "concern mapping" OR AB "map concern*" OR AB "mapping concern*" | 4 |
| 11 | AB "concern elicit*" OR AB "elicit concern*" OR AB "eliciting concern*" OR AB "elicited concern*" | 46 |
| 12 | AB "concern solicit*" OR AB "solicit concern*" OR AB "soliciting concern*" OR AB "solicited concern*" | 7 |
| 13 | AB "priority elicit*" OR AB "elicit priorit*" OR AB "eliciting priorit*" OR AB "elicited priorit*" | 7 |
| 14 | AB "priority solicit*" OR AB "solicit priorit*" OR AB "soliciting priorit*" OR AB "solicited priorit*" | 1 |
| 15 | AB "patient agenda*" OR AB "patient's agenda*" OR AB "patients' agenda*" | 52 |
| 16 | AB "opening statement*" | 89 |
| 17 | AB "visit opening*" OR AB "opening visit*" OR AB "encounter opening*" OR AB "opening encounter*" | 6 |
| 18 | AB "visit structur*" OR AB "structuring visit*" OR AB "structure visit*" OR AB "encounter structur*" OR AB "structuring encounter*" OR AB "structure encounter*" | 33 |
| 19 | S1 OR S2 OR S3 OR S4 OR S5 OR S6 OR S7 OR S8 OR S9 OR S10 OR S11 OR S12 OR S13 OR S14 OR S15 OR S16 OR S17 OR S18 | 1,201 |
| 20 | AB "patient*" OR AB "clinic*" | 1,179,845 |
| 21 | AB "approach*" OR AB "brochure*" OR AB "checklist*" OR AB "coach*" OR AB "conversation analys*" OR AB "conversation approach*" OR AB "dashboard*" OR AB "education*" OR AB "electronic health record*" OR AB "electronic medical record*" OR AB "form*" OR AB "framework*" OR AB "guide*" OR AB "guidance*" OR AB "handout*" OR AB "information*" OR AB "instrument*" OR AB "intervention*" OR AB "issue card*" OR AB "material*" OR AB "model*" OR AB "pamphlet*" OR AB "portal*" OR AB "prompt*" OR AB "protocol*" OR AB "question asking" OR AB "questionnaire*" OR AB "seminar*" OR AB "session*" OR AB "strateg*" OR AB "survey*" OR AB "technique*" OR AB "template*" OR AB "tool*" OR AB "training*" OR AB "webinar*" OR AB "workflow*" OR AB "worksheet*" OR AB "workshop*" | 3,383,966 |
| 22 | S19 AND S20 AND S21 | 209 |

**Database**: The Cochrane Library

**Dates covered**: CDSR, 1996 to present; CENTRAL, 1989 to present

**Date searched**: October 2023

**Limits**: None used.

**Search terms / results**: We searched titles, abstracts, and keywords in The Cochrane Library with the queries below. The search returned 110 results. We also include the breakdown by individual database in Table 4 below.

***Table 3.*** *The Cochrane Library search queries and results*

| **Search** | **Query** | **Results** |
| --- | --- | --- |
| 1 | ("agenda setting" OR (set NEXT agenda*) OR (setting NEXT agenda*)):ti,ab,kw | 71 |
| 2 | ("agenda mapping" OR (map NEXT agenda*) OR (mapping NEXT agenda*)):ti,ab,kw | 0 |
| 3 | ((agenda NEXT elicit*) OR (elicit NEXT agenda*) OR (eliciting NEXT agenda*) OR (elicited NEXT agenda*)):ti,ab,kw | 0 |
| 4 | ((agenda NEXT solicit*) OR (solicit NEXT agenda*) OR (soliciting NEXT agenda*) OR (solicited NEXT agenda*)):ti,ab,kw | 0 |
| 5 | (“topic setting” OR (set NEXT topic*) OR (setting NEXT topic*)):ti,ab,kw | 7 |
| 6 | (“topic mapping” OR (map NEXT topic*) OR (mapping NEXT topic*)):ti,ab,kw | 0 |
| 7 | ((topic NEXT elicit*) OR (elicit NEXT topic*) OR (eliciting NEXT topic*) OR (elicited NEXT topic*)):ti,ab,kw | 2 |
| 8 | ((topic NEXT solicit*) OR (solicit NEXT topic*) OR (soliciting NEXT topic*) OR (solicited NEXT topic*)):ti,ab,kw | 0 |
| 9 | (“concern setting” OR (set NEXT concern*) OR (setting NEXT concern*)):ti,ab,kw | 14 |
| 10 | (“concern mapping” OR (map NEXT concern*) OR (mapping NEXT concern*)):ti,ab,kw | 1 |
| 11 | ((concern NEXT elicit*) OR (elicit NEXT concern*) OR (eliciting NEXT concern*) OR (elicited NEXT concern*)):ti,ab,kw | 12 |
| 12 | ((concern NEXT solicit*) OR (solicit NEXT concern*) OR (soliciting NEXT concern*) OR (solicited NEXT concern*)):ti,ab,kw | 0 |
| 13 | ((priority NEXT elicit*) OR (elicit NEXT priorit*) OR (eliciting NEXT priorit*) OR (elicited NEXT priorit*)):ti,ab,kw | 0 |
| 14 | ((priority NEXT solicit*) OR (solicit NEXT priorit*) OR (soliciting NEXT priorit*) OR (solicited NEXT priorit*)):ti,ab,kw | 1 |
| 15 | ((patient NEXT agenda*) OR (patient’s NEXT agenda*) OR (patients’ NEXT agenda*)):ti,ab,kw | 17 |
| 16 | (opening NEXT statement*):ti,ab,kw | 1 |
| 17 | ((visit NEXT opening*) OR (opening NEXT visit*) OR (encounter NEXT opening*) OR (opening NEXT encounter*)):ti,ab,kw | 1 |
| 18 | ((visit NEXT structur*) OR (structuring NEXT visit*) OR (structure NEXT visit*) OR (encounter NEXT structur*) OR (structuring NEXT encounter*) OR (structure NEXT encounter*)):ti,ab,kw | 11 |
| 19 | #1 OR #2 OR #3 OR #4 OR #5 OR #6 OR #7 OR #8 OR #9 OR #10 OR #11 OR #12 OR #13 OR #14 OR #15 OR #16 OR #17 OR #18 | 131 |
| 20 | ((patient*) OR (clinic*)):ti,ab,kw | 1,512,530 |
| 21 | ((approach*) OR (brochure*) OR (checklist*) OR (coach*) OR (conversation NEXT analys*) OR (conversation NEXT approach*) OR (dashboard*) OR (education*) OR (electronic NEXT health NEXT record*) OR (electronic NEXT medical NEXT record*) OR (form*) OR (framework*) OR (guide*) OR (guidance*) OR (handout*) OR (information*) OR (instrument*) OR (intervention*) OR (issue NEXT card*) OR (material*) OR (model*) OR (pamphlet*) OR (portal*) OR (prompt*) OR (protocol*) OR “question asking” OR (questionnaire*) OR (seminar*) OR (session*) OR (strateg*) OR (survey*) OR (technique*) OR (template*) OR (tool*) OR (training*) OR (webinar*) OR (workflow*) OR (worksheet*) OR (workshop*)):ti,ab,kw | 1,213,926 |
| 22 | #19 AND #20 AND #21 | 110 |

***Table 4.*** *The Cochrane Library search results by database*

| **Database** | **Results** |
| --- | --- |
| Cochrane Reviews (Cochrane Database of Systematic Reviews) | 3 |
| Cochrane Protocols (Cochrane Database of Systematic Reviews) | 0 |
| Trials (Central Register of Controlled Trials) | 107 |
| Editorials | 0 |
| Special Collections | 0 |
| Clinical Answers | 0 |

**Database**: Cumulative Index to Nursing and Allied Health Literature (CINAHL)

**Dates covered**: 1981 to present

**Date searched**: October 2023

**Limits**: None used.

**Search terms / results**: We searched abstracts in CINAHL with the queries below. We were unable to identify any CINAHL Subject Headings for clinical visit agenda-setting. The search returned 320 results.

***Table 5.*** *CINAHL search queries and results*

| **Search** | **Query** | **Results** |
| --- | --- | --- |
| 1 | AB "agenda setting" OR AB "set agenda*" OR AB "setting agenda*" | 420 |
| 2 | AB "agenda mapping" OR AB "map agenda*" OR AB "mapping agenda*" | 2 |
| 3 | AB "agenda elicit*" OR AB "elicit agenda*" OR AB "eliciting agenda*" OR AB "elicited agenda*" | 4 |
| 4 | AB "agenda solicit*" OR AB "solicit agenda*" OR AB "soliciting agenda*" OR AB "solicited agenda*" | 2 |
| 5 | AB "topic setting" OR AB "set topic*" OR AB "setting topic*" | 230 |
| 6 | AB "topic mapping" OR AB "map topic*" OR AB "mapping topic*" | 16 |
| 7 | AB "topic elicit*" OR AB "elicit topic*" OR AB "eliciting topic*" OR AB "elicited topic*" | 6 |
| 8 | AB "topic solicit*" OR AB "solicit topic*" OR AB "soliciting topic*" OR AB "solicited topic*" | 1 |
| 9 | AB "concern setting" OR AB "set concern*" OR AB "setting concern*" | 39 |
| 10 | AB "concern mapping" OR AB "map concern*" OR AB "mapping concern*" | 1 |
| 11 | AB "concern elicit*" OR AB "elicit concern*" OR AB "eliciting concern*" OR AB "elicited concern*" | 35 |
| 12 | AB "concern solicit*" OR AB "solicit concern*" OR AB "soliciting concern*" OR AB "solicited concern*" | 4 |
| 13 | AB "priority elicit*" OR AB "elicit priorit*" OR AB "eliciting priorit*" OR AB "elicited priorit*" | 8 |
| 14 | AB "priority solicit*" OR AB "solicit priorit*" OR AB "soliciting priorit*" OR AB "solicited priorit*" | 1 |
| 15 | AB "patient agenda*" OR AB "patient's agenda*" OR AB "patients' agenda*" | 63 |
| 16 | AB "opening statement*" | 19 |
| 17 | AB "visit opening*" OR AB "opening visit*" OR AB "encounter opening*" OR AB "opening encounter*" | 6 |
| 18 | AB "visit structur*" OR AB "structuring visit*" OR AB "structure visit*" OR AB "encounter structur*" OR AB "structuring encounter*" OR AB "structure encounter*" | 31 |
| 19 | S1 OR S2 OR S3 OR S4 OR S5 OR S6 OR S7 OR S8 OR S9 OR S10 OR S11 OR S12 OR S13 OR S14 OR S15 OR S16 OR S17 OR S18 | 875 |
| 20 | AB "patient*" OR AB "clinic*" | 2,248,243 |
| 21 | AB "approach*" OR AB "brochure*" OR AB "checklist*" OR AB "coach*" OR AB "conversation analys*" OR AB "conversation approach*" OR AB "dashboard*" OR AB "education*" OR AB "electronic health record*" OR AB "electronic medical record*" OR AB "form*" OR AB "framework*" OR AB "guide*" OR AB "guidance*" OR AB "handout*" OR AB "information*" OR AB "instrument*" OR AB "intervention*" OR AB "issue card*" OR AB "material*" OR AB "model*" OR AB "pamphlet*" OR AB "portal*" OR AB "prompt*" OR AB "protocol*" OR AB "question asking" OR AB "questionnaire*" OR AB "seminar*" OR AB "session*" OR AB "strateg*" OR AB "survey*" OR AB "technique*" OR AB "template*" OR AB "tool*" OR AB "training*" OR AB "webinar*" OR AB "workflow*" OR AB "worksheet*" OR AB "workshop*" | 2,784,671 |
| 22 | S19 AND S20 AND S21 | 320 |

**Database**: MEDLINE via PubMed

**Dates covered**: 1946 to present

**Date searched**: October 2023

**Limits**: None used.

**Search terms / results**: We searched titles and abstracts in PubMed with the queries below. We were unable to identify any MeSH terms for clinical visit agenda-setting. The search returned 374 results.

***Table 6.*** *MEDLINE via PubMed search queries and results*

| **Search** | **Query** | **Results** |
| --- | --- | --- |
| 1 | "agenda setting"[Title/Abstract] OR "set agenda*"[Title/Abstract] OR "setting agenda*"[Title/Abstract] | 800 |
| 2 | "agenda mapping"[Title/Abstract] OR "map agenda*"[Title/Abstract] OR "mapping agenda*"[Title/Abstract] | 5 |
| 3 | "agenda elicit*"[Title/Abstract] OR "elicit agenda*"[Title/Abstract] OR "eliciting agenda*"[Title/Abstract] OR "elicited agenda*"[Title/Abstract] | 0 |
| 4 | "agenda solicit*"[Title/Abstract] OR "solicit agenda*"[Title/Abstract] OR "soliciting agenda*"[Title/Abstract] OR "solicited agenda*"[Title/Abstract] | 1 |
| 5 | "topic setting"[Title/Abstract] OR "set topic*"[Title/Abstract] OR "setting topic*"[Title/Abstract] | 12 |
| 6 | "topic mapping"[Title/Abstract] OR "map topic*"[Title/Abstract] OR "mapping topic*"[Title/Abstract] | 9 |
| 7 | "topic elicit*"[Title/Abstract] OR "elicit topic*"[Title/Abstract] OR "eliciting topic*"[Title/Abstract] OR "elicited topic*"[Title/Abstract] | 5 |
| 8 | "topic solicit*"[Title/Abstract] OR "solicit topic*"[Title/Abstract] OR "soliciting topic*"[Title/Abstract] OR "solicited topic*"[Title/Abstract] | 0 |
| 9 | "concern setting"[Title/Abstract] OR "set concern*"[Title/Abstract] OR "setting concern*"[Title/Abstract] | 0 |
| 10 | "concern mapping"[Title/Abstract] OR "map concern*"[Title/Abstract] OR "mapping concern*"[Title/Abstract] | 0 |
| 11 | "concern elicit*"[Title/Abstract] OR "elicit concern*"[Title/Abstract] OR "eliciting concern*"[Title/Abstract] OR "elicited concern*"[Title/Abstract] | 38 |
| 12 | "concern solicit*"[Title/Abstract] OR "solicit concern*"[Title/Abstract] OR "soliciting concern*"[Title/Abstract] OR "solicited concern*"[Title/Abstract] | 0 |
| 13 | "priority elicit*"[Title/Abstract] OR "elicit priorit*"[Title/Abstract] OR "eliciting priorit*"[Title/Abstract] OR "elicited priorit*"[Title/Abstract] | 0 |
| 14 | "priority solicit*"[Title/Abstract] OR "solicit priorit*"[Title/Abstract] OR "soliciting priorit*"[Title/Abstract] OR "solicited priorit*"[Title/Abstract] | 0 |
| 15 | "patient agenda*"[Title/Abstract] OR "patient's agenda*"[Title/Abstract] OR "patients' agenda*"[Title/Abstract] | 122 |
| 16 | "opening statement*"[Title/Abstract] | 64 |
| 17 | "visit opening*"[Title/Abstract] OR "opening visit*"[Title/Abstract] OR "encounter opening*"[Title/Abstract] OR "opening encounter*"[Title/Abstract] | 2 |
| 18 | "visit structur*"[Title/Abstract] OR "structuring visit*"[Title/Abstract] OR "structure visit*"[Title/Abstract] OR "encounter structur*"[Title/Abstract] OR "structuring encounter*"[Title/Abstract] OR "structure encounter*"[Title/Abstract] | 102 |
| 19 | #1 OR #2 OR #3 OR #4 OR #5 OR #6 OR #7 OR #8 OR #9 OR #10 OR #11 OR #12 OR #13 OR #14 OR #15 OR #16 OR #17 OR #18 | 1,143 |
| 20 | "patient*"[Title/Abstract] OR "clinic*"[Title/Abstract] | 10,492,620 |
| 21 | "approach*"[Title/Abstract] OR "brochure*"[Title/Abstract] OR "checklist*"[Title/Abstract] OR "coach*"[Title/Abstract] OR "conversation analys*"[Title/Abstract] OR "conversation approach*"[Title/Abstract] OR "dashboard*"[Title/Abstract] OR "education*"[Title/Abstract] OR "electronic health record*"[Title/Abstract] OR "electronic medical record*"[Title/Abstract] OR "form*"[Title/Abstract] OR "framework*"[Title/Abstract] OR "guide*"[Title/Abstract] OR "guidance*"[Title/Abstract] OR "handout*"[Title/Abstract] OR "information*"[Title/Abstract] OR "instrument*"[Title/Abstract] OR "intervention*"[Title/Abstract] OR "issue card*"[Title/Abstract] OR "material*"[Title/Abstract] OR "model*"[Title/Abstract] OR "pamphlet*"[Title/Abstract] OR "portal*"[Title/Abstract] OR "prompt*"[Title/Abstract] OR "protocol*"[Title/Abstract] OR "question asking"[Title/Abstract] OR "questionnaire*"[Title/Abstract] OR "seminar*"[Title/Abstract] OR "session*"[Title/Abstract] OR "strateg*"[Title/Abstract] OR "survey*"[Title/Abstract] OR "technique*"[Title/Abstract] OR "template*"[Title/Abstract] OR "tool*"[Title/Abstract] OR "training*"[Title/Abstract] OR "webinar*"[Title/Abstract] OR "workflow*"[Title/Abstract] OR "worksheet*"[Title/Abstract] OR "workshop*"[Title/Abstract] | 15,190,081 |
| 22 | #19 AND #20 AND #21 | 374 |

**Database**: ProQuest

**Dates covered**: 1861 to present

**Date searched**: October 2023

**Limits**: None used.

**Search terms / results**: We searched abstracts and summaries in ProQuest with the queries below. The search returned 374 results.

***Table 7.*** *ProQuest search queries and results*

| **Search** | **Query** | **Results** |
| --- | --- | --- |
| 1 | summary(“agenda setting” OR “set agenda” OR “set agendas” OR “setting agenda” OR “set agendas”) | 5,461 |
| 2 | summary(“agenda mapping” OR “map agenda” OR “map agendas” OR “mapping agenda” OR “mapping agendas”) | 11 |
| 3 | summary(“agenda elicitation” OR “elicit agenda” OR “elicit agendas” OR “eliciting agenda” OR “eliciting agendas” OR “elicited agenda” OR “elicited agendas”) | 2 |
| 4 | summary(“agenda solicitation” OR “solicit agenda” OR “solicit agendas” OR “soliciting agenda” OR “soliciting agendas” OR “solicited agenda” OR “solicited agendas”) | 3 |
| 5 | summary(“topic setting” OR “set topic” OR “set topics” OR “setting topic” OR “setting topics”) | 157 |
| 6 | summary(“topic mapping” OR “map topic” OR “map topics” OR “mapping topic” OR “mapping topics”) | 71 |
| 7 | summary(“topic elicitation” OR “elicit topic” OR “elicit topics” OR “eliciting topic” OR “eliciting topics” OR “elicited topic” OR “elicited topics”) | 9 |
| 8 | summary(“topic solicitation” OR “solicit topic” OR “solicit topics” OR “soliciting topic” OR “soliciting topics” OR “solicited topic” OR “solicited topics”) | 9 |
| 9 | summary(“concern setting” OR “set concern” OR “set concerns” OR “setting concern” OR “setting concerns”) | 112 |
| 10 | summary(“concern mapping” OR “map concern” OR “map concerns” OR “mapping concern” OR “mapping concerns”) | 35 |
| 11 | summary(“concern elicitation” OR “elicit concern” OR “elicit concerns” OR “eliciting concern” OR “eliciting concerns” OR “elicited concern” OR “elicited concerns”) | 224 |
| 12 | summary(“concern solicitation” OR “solicit concern” OR “solicit concerns” OR “soliciting concern” OR “soliciting concerns” OR “solicited concern” OR “solicited concerns”) | 32 |
| 13 | summary(“priority elicitation” OR “elicit priority” OR “elicit priorities” OR “eliciting priority” OR “eliciting priorities” OR “elicited priority” OR “elicited priorities”) | 15 |
| 14 | summary(“priority solicitation” OR “solicit priority” OR “solicit priorities” OR “soliciting priority” OR “soliciting priorities” OR “solicited priority” OR “solicited priorities”) | 3 |
| 15 | summary(“patient agenda” OR “patient agendas” OR “patient’s agenda” OR “patient’s agendas” OR “patients’ agenda” OR “patients’ agendas”) | 97 |
| 16 | summary(“opening statement” OR “opening statements”) | 41,737 |
| 17 | summary(“visit opening” OR “visit openings” OR “opening visit” OR “opening visits” OR “encounter opening” OR “encounter openings” OR “opening encounter” OR “opening encounters”) | 1,088 |
| 18 | summary(“visit structure” OR “visit structures” OR “visit structuring” OR “visit structured” OR “structuring visit” OR “structuring visits” OR “structure visit” OR “structure visits” OR “encounter structure” OR “encounter structures” OR “encounter structuring” OR “encounter structured” OR “structuring encounter” OR “structuring encounters” OR “structure encounter” OR “structure encounters”) | 104 |
| 19 | [S1] OR [S2] OR [S3] OR [S4] OR [S5] OR [S6] OR [S7] OR [S8] OR [S9] OR [S10] OR [S11] OR [S12] OR [S13] OR [S14] OR [S15] OR [S16] OR [S17] OR [S18] | 49,156 |
| 20 | summary(patient OR patients OR patient’s OR patients’ OR clinic OR clinical OR clinician OR clinicians) | 7,871,002 |
| 21 | summary(approach OR approaches OR brochure OR brochures OR checklist OR checklists OR coach OR coaching OR “conversation analysis” OR “conversation analyses” OR “conversation approach” OR “conversation approaches” OR dashboard OR dashboards OR education OR educational OR “electronic health record” OR “electronic health records” OR “electronic medical record” OR “electronic medical records” OR form OR forms OR framework OR frameworks OR guide OR guides OR guidance OR handout OR handouts OR information OR informational OR instrument OR instruments OR intervention OR interventions OR “issue card” OR “issue cards” OR material OR materials OR model OR models OR modeling OR pamphlet OR pamphlets OR portal OR portals OR prompt OR prompts OR prompting OR protocol OR protocols OR “question asking” OR questionnaire OR questionnaires OR seminar OR seminars OR session OR sessions OR strategy OR strategies OR survey OR surveys OR technique OR techniques OR template OR templates OR tool OR tools OR training OR trainings OR trained OR webinar OR webinars OR workflow OR workflows OR worksheet OR worksheets OR workshop OR workshops) | 60,655,883 |
| 22 | [S19] AND [S20] AND [S21] | 374 |

**Database**: Scopus

**Dates covered**: 1788 to present

**Date searched**: October 2023

**Limits**: None used.

**Search terms / results**: We searched titles, abstracts, and keywords in Scopus with the queries below. The search returned 1,023 results.

***Table 8.*** *Scopus search queries and results*

| **Search** | **Query** | **Results** |
| --- | --- | --- |
| 1 | TITLE-ABS-KEY ( "agenda setting" OR "set* agenda*" ) | 5,776 |
| 2 | TITLE-ABS-KEY ( "agenda mapping" OR "map* agenda*" ) | 14 |
| 3 | TITLE-ABS-KEY ( "agenda elicit*" OR "elicit* agenda*" ) | 7 |
| 4 | TITLE-ABS-KEY ( "agenda solicit*" OR "solicit* agenda*" ) | 3 |
| 5 | TITLE-ABS-KEY ( "topic setting" OR "set* topic*" ) | 261 |
| 6 | TITLE-ABS-KEY ( "topic mapping" OR "map* topic*" ) | 157 |
| 7 | TITLE-ABS-KEY ( "topic elicit*" OR "elicit* topic*" ) | 67 |
| 8 | TITLE-ABS-KEY ( "topic solicit*" OR "solicit* topic*" ) | 8 |
| 9 | TITLE-ABS-KEY ( "concern setting" OR "set* concern*" ) | 972 |
| 10 | TITLE-ABS-KEY ( "concern mapping" OR "map* concern*" ) | 255 |
| 11 | TITLE-ABS-KEY ( "concern elicit*" OR "elicit* concern*" ) | 222 |
| 12 | TITLE-ABS-KEY ( "concern solicit*" OR "solicit* concern*" ) | 23 |
| 13 | TITLE-ABS-KEY ( "priority elicit*" OR "elicit* priorit*" ) | 50 |
| 14 | TITLE-ABS-KEY ( "priority solicit*" OR "solicit* priorit*" ) | 4 |
| 15 | TITLE-ABS-KEY ( "patient* agenda*" ) | 145 |
| 16 | TITLE-ABS-KEY ( "opening statement*" ) | 585 |
| 17 | TITLE-ABS-KEY ( "visit opening*" OR "opening visit*" OR "encounter opening*" OR "opening encounter*" ) | 29 |
| 18 | TITLE-ABS-KEY ( "visit structur*" OR "structur* visit*" OR "encounter structur*" OR "structur* encounter*" ) | 846 |
| 19 | #1 OR #2 OR #3 OR #4 OR #5 OR #6 OR #7 OR #8 OR #9 OR #10 OR #11 OR #12 OR #13 OR #14 OR #15 OR #16 OR #17 OR #18 | 9,388 |
| 20 | TITLE-ABS-KEY ( "patient*" OR "clinic*" ) | 15,942,003 |
| 21 | TITLE-ABS-KEY ( "approach*" OR "brochure*" OR "checklist*" OR "coach*" OR "conversation analys*" OR "conversation* approach*" OR "dashboard*" OR "education*" OR "electronic health record*" OR "electronic medical record*" OR "form*" OR "framework*" OR "guide*" OR "guidance*" OR "handout*" OR "information*" OR "instrument*" OR "intervention*" OR "issue card*" OR "material*" OR "model*" OR "pamphlet*" OR "portal*" OR "prompt*" OR "protocol*" OR "question asking" OR "questionnaire*" OR "seminar*" OR "session*" OR "strateg*" OR "survey*" OR "technique*" OR "template*" OR "tool*" OR "training*" OR "webinar*" OR "workflow*" OR "worksheet*" OR "workshop*" ) | 50,838,378 |
| 22 | #19 AND #20 AND #21 | 1,023 |

**Database**: Web of Science

**Dates covered**: 1900 to present

**Date searched**: October 2023

**Limits**: None used.

**Search terms / results**: We searched abstracts in Web of Science with the queries below. The search returned 562 results.

***Table 9.*** *Web of Science search queries and results*

| **Search** | **Query** | **Results** |
| --- | --- | --- |
| 1 | AB=("agenda setting" OR "set* agenda*") | 3,470 |
| 2 | AB=("agenda mapping" OR "map* agenda*") | 5 |
| 3 | AB=("agenda elicit*" OR "elicit* agenda*") | 5 |
| 4 | AB=("agenda solicit*" OR "solicit* agenda*") | 2 |
| 5 | AB=("topic setting" OR "set* topic*") | 129 |
| 6 | AB=("topic mapping" OR "map* topic*") | 73 |
| 7 | AB=("topic elicit*" OR "elicit* topic*") | 28 |
| 8 | AB=("topic solicit*" OR "solicit* topic*") | 4 |
| 9 | AB=("concern setting" OR "set* concern*") | 703 |
| 10 | AB=("concern mapping" OR "map* concern*") | 126 |
| 11 | AB=("concern elicit*" OR "elicit* concern*") | 140 |
| 12 | AB=("concern solicit*" OR "solicit* concern*") | 16 |
| 13 | AB=("priority elicit*" OR "elicit* priorit*") | 42 |
| 14 | AB=("priority solicit*" OR "solicit* priorit*") | 2 |
| 15 | AB=("patient* agenda*") | 58 |
| 16 | AB=("opening statement*") | 113 |
| 17 | AB=("visit opening*" OR "opening visit*" OR "encounter opening*" OR "opening encounter*") | 13 |
| 18 | AB=("visit structur*" OR "structur* visit*" OR "encounter structur*" OR "structur* encounter*") | 572 |
| 19 | #1 OR #2 OR #3 OR #4 OR #5 OR #6 OR #7 OR #8 OR #9 OR #10 OR #11 OR #12 OR #13 OR #14 OR #15 OR #16 OR #17 OR #18 | 5,480 |
| 20 | AB=("patient*" OR "clinic*") | 8,185,988 |
| 21 | AB=("approach*" OR "brochure*" OR "checklist*" OR "coach*" OR "conversation analys*" OR "conversation* approach*" OR "dashboard*" OR "education*" OR "electronic health record*" OR "electronic medical record*" OR "form*" OR "framework*" OR "guide*" OR "guidance*" OR "handout*" OR "information*" OR "instrument*" OR "intervention*" OR "issue card*" OR "material*" OR "model*" OR "pamphlet*" OR "portal*" OR "prompt*" OR "protocol*" OR "question asking" OR "questionnaire*" OR "seminar*" OR "session*" OR "strateg*" OR "survey*" OR "technique*" OR "template*" OR "tool*" OR "training*" OR "webinar*" OR "workflow*" OR "worksheet*" OR "workshop*") | 30,805,720 |
| 22 | #19 AND #20 AND #21 | 562 |

### Trial registry searches

**Database**: ClinicalTrials.gov

**Dates covered**: 1997 to present

**Date searched**: October 2023

**Limits**: None used.

**Search terms / results**: We searched ClinicalTrials.gov using the search string below entered in the ‘Other terms’ field. The search returned 20 results.

*"agenda setting" OR "agenda mapping" OR "patient agenda" OR "visit opening" OR "topic elicitation" OR "topic solicitation"*

### Other searches

**Source**: Google Scholar

**Dates covered**: Unknown

**Date searched:** October 2023

**Limits**: None used.

**Search terms / results**: We searched Google Scholar using the search string below, without using any modifiers. The search returned about 18,500 results.

*("agenda setting" OR "agenda mapping" OR "patient agenda" OR "visit opening" OR "topic elicitation" OR "topic solicitation") AND clinic* AND intervention**
