# Supplementary material for: Agenda-setting in the clinical encounter: A systematic review protocol
Source: PLoS One. 2024 Oct 24;19(10):e0312613. doi: 10.1371/journal.pone.0312613 (PMC11500969; doi:10.1371/journal.pone.0312613)
Supplement: S3 File — (DOCX) [file pone.0312613.s003.docx]

## Supporting Information 3. Summary of inclusion criteria and agenda-setting definitions

***Table 1.*** *Summary of preliminary study inclusion criteria*

| **Order** | **Category** | **Inclusion criterion** |
| --- | --- | --- |
| 1 | Study design | Include all randomized trials comparing an agenda-setting intervention to either no intervention (usual care) or to at least one other agenda-setting intervention. Include non-randomized trials only if there is also a comparison (pre-post or quasi-experimental designs). Include feasibility studies and pilot trials. Exclude non-comparative designs and qualitative studies. |
| 2 | Population | Include all participants and clinical settings. |
| 3 | Intervention | Include all interventions specifically aiming to promote or increase clinical visit agenda-setting. Exclude interventions only incidentally involving agenda-setting. **Agenda-setting is a practice in which a clinician works collaboratively with a patient to elicit, and often propose or organize, the topics to be discussed during a clinical encounter.**[[1–6]](https://paperpile.com/c/roWcVC/1F3no+Z4DKB+3GfwU+KtCpF+hgSaU+mQ3fW) **It must occur before or at the start of the encounter, but does not need to be performed to exhaustion.**[[2,4,7,8]](https://paperpile.com/c/roWcVC/KIALa+17hoD+Z4DKB+KtCpF) |
| 4 | Outcomes | Include all study-specified primary and secondary outcomes. Include all units of measurement used in these outcomes. |

***Table 2.*** *Development of our working definition of clinical visit agenda-setting based on existing definitions in the literature*

| **Definition of agenda-setting** | **Comment** |
| --- | --- |
| “Setting an agenda (i.e. a set of discussion topics of importance to the patient or the clinician to be covered in the encounter)” with a patient”[[1]](https://paperpile.com/c/roWcVC/1F3no) | Topics may be important to **either the patient or clinician** |
| “Agenda-mapping is a collaborative communication strategy that enables clinicians and patients to establish shared focus on what to talk about by mapping options and agreeing priorities”[[2]](https://paperpile.com/c/roWcVC/Z4DKB) | Covers topic **organization**, emphasizes equal **collaboration** |
| “Patients talk about their concerns, wishes, requests, and/or goals, clinicians raise subjects they consider important, [...] clinicians and patients agree shared priorities, a focus of what to talk about during the session is agreed, the conversation is collaborative, patients are involved and engaged in the conversation, a broad overview of potential discussion topics is constructed, the rest of of the consultation is structured based on the shared agenda”[[3]](https://paperpile.com/c/roWcVC/3GfwU) | Topics are raised by **either the patient or clinician**, engaging in **collaborative identification** and **prioritization** of topics |
| “To what extent did the clinician attempt to identify all possible talk topics upfront [...] and prioritize and agree a shared focus?”[[2,4]](https://paperpile.com/c/roWcVC/Z4DKB+KtCpF) | Items are from a validated measure of agenda-mapping (EAGL-I), covers topic **elicitation** and **organization**, must be **collaborative**, and occur at the **start of the encounter** |
| “A communication strategy physicians use at the beginning of clinical visits to elicit, propose, and organize a complete list of topics to be covered”[[7]](https://paperpile.com/c/roWcVC/KIALa) | Covers topic **elicitation** and **organization**, must occur at the **start of the encounter** |
| “The ability of patients and their physicians to work together to identify and align priorities for a visit”[[5]](https://paperpile.com/c/roWcVC/hgSaU) | **Identification** and **prioritization** of topics, **collaborative** |
| “Soliciting patients’ chief complaints with open-ended question formats such as “What can I do for you?” or “How can I help?” [...] This type of agenda pursuit has its greatest effects when performed ‘early’ or ‘up front’ during visits”[[8]](https://paperpile.com/c/roWcVC/17hoD) | Emphasizes **soliciting** topics, should occur at the **start of the encounter** |
| “Agenda setting is a strategy that seeks to improve the effectiveness of patient-clinician interactions by establishing relational “ground rules,” identifying priorities, and negotiating conversational focus”[[6]](https://paperpile.com/c/roWcVC/mQ3fW) | **Identification** and **organization** of topics, establishing **patient-clinician relationship** |

#### References

1. [Allgood S, Park J, Soleiman K, Saha S, Han D, McArthur A, et al. Taxonomy and effectiveness of clinician agenda-setting questions in routine ambulatory encounters: A mixed method study. Patient Educ Couns. 2023;115: 107889. doi:](http://paperpile.com/b/roWcVC/1F3no)[10.1016/j.pec.2023.107889](http://dx.doi.org/10.1016/j.pec.2023.107889)

2. [Gobat N, Kinnersley P, Gregory JW, Pickles T, Hood K, Robling M. Measuring clinical skills in agenda-mapping (EAGL-I). Patient Educ Couns. 2015;98: 1214–1221. doi:](http://paperpile.com/b/roWcVC/Z4DKB)[10.1016/j.pec.2015.06.018](http://dx.doi.org/10.1016/j.pec.2015.06.018)

3. [Gobat N, Kinnersley P, Gregory JW, Robling M. What is agenda setting in the clinical encounter? Consensus from literature review and expert consultation. Patient Educ Couns. 2015;98: 822–829. doi:](http://paperpile.com/b/roWcVC/3GfwU)[10.1016/j.pec.2015.03.024](http://dx.doi.org/10.1016/j.pec.2015.03.024)

4. [Coding manual. Evaluation of AGenda mapping skilL -­‐ Instrument (EAGL-­‐I v1.5.1). [cited 21 Aug 2023]. Available:](http://paperpile.com/b/roWcVC/KtCpF) <https://orca.cardiff.ac.uk/id/eprint/73161/1/EAGL-I%20v1.5.1%20manual%20May15.pdf>

5. [Kowalski CP, McQuillan DB, Chawla N, Lyles C, Altschuler A, Uratsu CS, et al. “The Hand on the Doorknob”: Visit Agenda Setting by Complex Patients and Their Primary Care Physicians. J Am Board Fam Med. 2018;31: 29–37. doi:](http://paperpile.com/b/roWcVC/hgSaU)[10.3122/jabfm.2018.01.170167](http://dx.doi.org/10.3122/jabfm.2018.01.170167)

6. [Wolff JL, Roter DL, Boyd CM, Roth DL, Echavarria DM, Aufill J, et al. Patient–Family Agenda Setting for Primary Care Patients with Cognitive Impairment: the SAME Page Trial. J Gen Intern Med. 2018;33: 1478–1486. doi:](http://paperpile.com/b/roWcVC/mQ3fW)[10.1007/s11606-018-4563-y](http://dx.doi.org/10.1007/s11606-018-4563-y)

7. [Hood-Medland EA, White AEC, Kravitz RL, Henry SG. Agenda setting and visit openings in primary care visits involving patients taking opioids for chronic pain. BMC Fam Pract. 2021;22: 4. doi:](http://paperpile.com/b/roWcVC/KIALa)[10.1186/s12875-020-01317-4](http://dx.doi.org/10.1186/s12875-020-01317-4)

8. [Robinson JD, Tate A, Heritage J. Agenda-setting revisited: When and how do primary-care physicians solicit patients’ additional concerns? Patient Educ Couns. 2016;99: 718–723. doi:](http://paperpile.com/b/roWcVC/17hoD)[10.1016/j.pec.2015.12.009](http://dx.doi.org/10.1016/j.pec.2015.12.009)
