# Supplementary material for: Agenda-setting in the clinical encounter: A systematic review protocol
Source: PLoS One. 2024 Oct 24;19(10):e0312613. doi: 10.1371/journal.pone.0312613 (PMC11500969; doi:10.1371/journal.pone.0312613)
Supplement: Attachment — Submitted filename: PONE-D-24-12064 (1).pdf Reviewed Sabit.pdf [file pone.0312613.s004.pdf]

PLOS ONE

Agenda-setting in the clinical encounter: a systematic review protocol

--Manuscript Draft--

**Manuscript Number:**	PONE-D-24-12064
**Article Type:**	Study Protocol
**Full Title:**	Agenda-setting in the clinical encounter: a systematic review protocol
**Short Title:**	Agenda-setting in the clinical encounter
**Corresponding Author:**	Catherine Saunders<br>Dartmouth Health<br>Thetford Center, Vermont UNITED STATES
**Keywords:**	agenda-setting; agenda setting; patient-centered communication; clinical encounter; clinical visit; visit structuring; topic elicitation; topic solicitation; visit agenda
**Abstract:**	Introduction<br>Agenda-setting is a collaborative communication strategy used by a clinician before or at the start of a clinical encounter to work together with the patient to “elicit, propose, and organize” topics to be discussed during the encounter. While agenda-setting has been acknowledged as an important element of patient-centered communication, the effectiveness of agenda-setting interventions in improving healthcare outcomes is unclear. To our knowledge, no systematic review has examined clinical visit agenda-setting interventions.<br>Methods and analysis<br>The primary aim of the systematic review will be to assess the effects of agenda-setting interventions on outcomes relating to the clinical encounter itself, patients, and clinicians, as well as any other study-specified outcomes. Our secondary aims will be to examine the characteristics and delivery attributes of agenda-setting interventions, as well as how agenda-setting has been operationalized and measured. We will search selected databases and gray literature from inception until October 2023. All studies comparing a clinical visit agenda-setting intervention with either usual care or another agenda-setting intervention will be included. Two independent reviewers will complete article screening and data extraction, with a third independent reviewer resolving any conflicts. We will assess all studies’ methodological quality and the quality of their evidence using standardized criteria. If a sufficient number of studies report the same outcomes, we will pool their results and perform a meta-analysis of those outcomes. We will also synthesize all results qualitatively, regardless of whether we are able to complete a meta-analysis.<br>PROSPERO registration number<br>CRD42023468045
**Order of Authors:**	Ailyn Sierpe, MSc
	Renata W. Yen, PhD, MPH
	Gabrielle Stevens, PhD
	Aricca D. Van Citters, MS
	Glyn Elwyn, MD, PhD, MSc
	Catherine H. Saunders, PhD, MPH
**Additional Information:**	
**Question**	**Response**
**Financial Disclosure**<br><br>Enter a financial disclosure statement that describes the sources of funding for the work included in this submission. Review the submission guidelines for detailed	The author(s) received no specific funding for this work.

Powered by Editorial Manager® and ProduXion Manager® from Aries Systems Corporation

requirements. View published research articles from *PLOS ONE* for specific examples.<br><br>This statement is required for submission and **will appear in the published article** if the submission is accepted. Please make sure it is accurate.<br><br>**Funded studies**<br>Enter a statement with the following details:<br>• Initials of the authors who received each award<br>• Grant numbers awarded to each author<br>• The full name of each funder<br>• URL of each funder website<br>• Did the sponsors or funders play any role in the study design, data collection and analysis, decision to publish, or preparation of the manuscript?<br><br>Did you receive funding for this work?	
**Competing Interests**<br><br>Use the instructions below to enter a competing interest statement for this submission. On behalf of all authors, disclose any competing interests that could be perceived to bias this work—acknowledging all financial support and any other relevant financial or non-financial competing interests.<br><br>This statement is required for submission and **will appear in the published article** if the submission is accepted. Please make sure it is accurate and that any funding sources listed in your Funding Information later in the submission form are also declared in your Financial Disclosure statement.<br><br>View published research articles from *PLOS ONE* for specific examples.	Ailyn Sierpe:<br>No conflicts of interest.<br><br>Renata Yen:<br>No conflicts of interest.<br><br>Gabrielle Stevens:<br>I have co-authored a published conference abstract about the feasibility of a goal-based agenda-setting intervention (citation below). This publication may appear in search results for this review, but will not meet eligibility criteria due to the lack of a comparison group.<br>Stevens G, Elwyn G. Feasibility of a goal-based agenda setting intervention for informing conversations in adult cystic fibrosis care: The goal talk study. Journal of Cystic Fibrosis. 2021. https://www.ScienceDirect.com/journal/journal-of-cystic-fibrosis/vol/20/suppl/S2<br>I have also co-authored a publication about the evaluation of a question prompt intervention to increase shared-decision making in clinical encounters (citation below). This publication may appear in search results for this review, but will likely not meet eligibility criteria due to the study focus.<br>Thompson R, Stevens G, Manski R, Donnelly KZ, Agusti D, Li Z, et al. Right For Me: a pragmatic multi-arm cluster randomised controlled trial of two interventions for increasing shared decision-making about contraceptive methods. medRxiv. 2021. https://www.medrxiv.org/content/10.1101/2021.06.25.21257891v1<br>Additionally, I have a broad research interest in the development and evaluation of agenda-setting interventions, have received funding for these purposes in the past, and may again in the future.

Powered by Editorial Manager® and ProduXion Manager® from Aries Systems Corporation

**NO authors have competing interests**<br><br>Enter: *The authors have declared that no competing interests exist.*<br><br>**Authors with competing interests**<br><br>Enter competing interest details beginning with this statement:<br><br>*I have read the journal's policy and the authors of this manuscript have the following competing interests: [insert competing interests here]*<br><br><br><br>* typeset	Aricca Van Citters:<br>I have co-authored several publications that include agenda-setting as a component of a larger intervention (citations below), and am conducting research on the types of concerns that agenda-setting questions elicit and their impact on shared decision-making. These publications may appear in search results for this review, but will likely not meet eligibility criteria due to the study focus.<br>Van Citters AD, Gifford AH, Brady C, Dunitz JM, Elmhirst M, Flath J, Laguna TA, Moore B, Prickett ML, Riordan M, Savant AP, Gore W, Jian S, Soper M, Marshall BC, Nelson EC, Sabadosa KA. Formative evaluation of a dashboard to support coproduction of healthcare services in cystic fibrosis. J Cyst Fibros. 2020;19(5):768-76. Epub 2020/05/02. doi: 10.1016/j.jcf.2020.03.009. PubMed PMID: 32354650.<br>Van Citters AD, Holthoff MM, Kennedy AM, Melmed GY, Oberai R, Siegel CA, Weaver A, Nelson EC. Point-of-care dashboards promote coproduction of healthcare services for patients with inflammatory bowel disease. Int J Qual Health Care. 2021;33(Supplement_2):ii40-ii7. doi: 10.1093/intqhc/mzab067. PubMed PMID: 34849970.<br>Van Citters AD, Taxter AJ, Mathew SD, Lawson E, Eseddi J, Del Gaizo V, Ahmad J, Bajaj P, Courtnay S, Davila L, Donaldson B, Kimura Y, Lee T, Mecchella JN, Nelson EC, Pompa S, Tabussi D, Johnson LC. Enhancing Care Partnerships Using a Rheumatology Dashboard: Bringing Together What Matters Most to Both Patients and Clinicians. ACR Open Rheumatol. 2023. Epub 2023/03/01. doi: 10.1002/acr2.11533. PubMed PMID: 36852527.<br>I have also received grant funding to test the feasibility of agenda-setting interventions, as components of larger studies.<br><br>Glyn Elwyn:<br>I have co-authored a published conference abstract about the feasibility of a goal-based agenda-setting intervention (citation below). This publication may appear in search results for this review, but will not meet eligibility criteria due to the lack of a comparison group.<br>Stevens G, Elwyn G. Feasibility of a goal-based agenda setting intervention for informing conversations in adult cystic fibrosis care: The goal talk study. Journal of Cystic Fibrosis. 2021. https://www.ScienceDirect.com/journal/journal-of-cystic-fibrosis/vol/20/suppl/S2<br>I have also co-authored a publication about the evaluation of a question prompt intervention to increase shared-decision making in clinical encounters (citation below). This publication may appear in search results for this review, but will likely not meet eligibility criteria due to the study focus.<br>Thompson R, Stevens G, Manski R, Donnelly KZ, Agusti D, Li Z, et al. Right For Me: a pragmatic multi-arm cluster randomised controlled trial of two interventions for increasing shared decision-making about contraceptive methods. medRxiv. 2021. https://www.medrxiv.org/content/10.1101/2021.06.25.21257891v1<br>I have also co-authored a publication about the development of a clinical visit agenda-setting tool, called Serious Illness Topics (citation below). This publication may appear in search results for this review, but will likely not meet eligibility criteria due to study design.<br>Saunders CH, Durand M-A, Scalia P, Kirkland KB, MacMartin MA, Barnato AE, et al. 'It helps us say what's important...' Developing Serious Illness Topics: A clinical visit agenda-setting tool. Patient Educ Couns 2023;113:107764. https://doi.org/10.1016/j.pec.2023.107764.<br>Additionally, I have a broad research interest in the development and evaluation of agenda-setting interventions, have received funding for these purposes in the past,

Powered by Editorial Manager® and ProduXion Manager® from Aries Systems Corporation

	and may again in the future.<br><br>Catherine Saunders:<br>I have co-authored a publication about the development of a clinical visit agenda-setting tool, called Serious Illness Topics (citation below). This publication may appear in search results for this review, but will likely not meet eligibility criteria due to study design.<br>Saunders CH, Durand M-A, Scalia P, Kirkland KB, MacMartin MA, Barnato AE, et al. 'It helps us say what's important...' Developing Serious Illness Topics: A clinical visit agenda-setting tool. Patient Educ Couns 2023;113:107764. https://doi.org/10.1016/j.pec.2023.107764.<br>Additionally, I have received funding to observe naturally-occurring clinical visit agenda-setting, although the research has not yet begun and the findings have not yet been published. I have a research interest in clinical visit agenda-setting and will likely publish on this topic in the future.
**Ethics Statement**<br><br>Enter an ethics statement for this submission. This statement is required if the study involved:<br><br>• Human participants<br>• Human specimens or tissue<br>• Vertebrate animals or cephalopods<br>• Vertebrate embryos or tissues<br>• Field research<br><br>Write "N/A" if the submission does not require an ethics statement.<br><br>General guidance is provided below. Consult the submission guidelines for detailed instructions. **Make sure that all information entered here is included in the Methods section of the manuscript.**	N/A

Powered by Editorial Manager® and ProduXion Manager® from Aries Systems Corporation

**Format for specific study types**<br><br>**Human Subject Research (involving human participants and/or tissue)**<br>• Give the name of the institutional review board or ethics committee that approved the study<br>• Include the approval number and/or a statement indicating approval of this research<br>• Indicate the form of consent obtained (written/oral) or the reason that consent was not obtained (e.g. the data were analyzed anonymously)<br><br>**Animal Research (involving vertebrate animals, embryos or tissues)**<br>• Provide the name of the Institutional Animal Care and Use Committee (IACUC) or other relevant ethics board that reviewed the study protocol, and indicate whether they approved this research or granted a formal waiver of ethical approval<br>• Include an approval number if one was obtained<br>• If the study involved *non-human primates*, add *additional details* about animal welfare and steps taken to ameliorate suffering<br>• If anesthesia, euthanasia, or any kind of animal sacrifice is part of the study, include briefly which substances and/or methods were applied<br><br>**Field Research**<br><br>Include the following details if this study involves the collection of plant, animal, or other materials from a natural setting:<br>• Field permit number<br>• Name of the institution or relevant body that granted permission	
**Data Availability**<br><br>Authors are required to make the data underlying their research findings fully available, without restriction.<br><br>If the manuscript reports pilot or preliminary results, the data underlying those results must be made available at the time of publication. A Data Availability	N/A - No results are reported

Powered by Editorial Manager® and ProduXion Manager® from Aries Systems Corporation

Statement describing where the data can be found is required at submission. Your answers to this question constitute the Data Availability Statement and will be published in the article, if accepted. When legal or ethical requirements prohibit public data sharing, authors must indicate how researchers may obtain access to the data. See the PLOS Data Policy and FAQ for detailed information.<br><br>**Important:** Stating 'data available on request from the author' is not sufficient. If your data are only available upon request, select 'No' for the first question and explain your situation in the text box.<br><br>For protocols without pilot or preliminary data, authors are strongly encouraged to state how they plan to share research data from their study when it is completed or published.<br><br>If your study includes pilot data, will they be made fully available without restriction?	
**Describe where the data may be found in full sentences. If you are copying our sample text, replace any instances of XXX with the appropriate details.**<br><br>• If **no pilot data are reported** in the manuscript, please enter the following: *No datasets were generated or analysed during the current study. All relevant data from this study will be made available upon study completion.*<br>• If the manuscript reports pilot data **held in a public repository**, include URLs, accession numbers or DOIs. If this information will only be available after acceptance, indicate this by ticking the box below. For example: *All XXX files are available from the XXX database (accession number(s) XXX, XXX.).*<br>• If the manuscript reports pilot data contained **within the manuscript and/or**	No datasets were generated or analyzed in the preparation of this protocol. All relevant data will be made available upon study completion.

**Supporting Information files**, enter the following:<br>*All relevant data are within the manuscript and its Supporting Information files.*<br>• If neither of these applies but you are able to provide **details of access elsewhere**, with or without limitations, please do so. For example:<br><br>*Data cannot be shared publicly because of [XXX]. Data are available from the XXX Institutional Data Access / Ethics Committee (contact via XXX) for researchers who meet the criteria for access to confidential data.*<br><br>*The data underlying the results presented in the study are available from (include the name of the third party and contact information or URL).*<br>• This text is appropriate if the data are owned by a third party and authors do not have permission to share the data.<br><br>For study protocols with data management or sharing plans, authors are encouraged to briefly describe in their Data Availability Statement how data they generate will be made accessible when the study is completed. For example:<br>• Deidentified research data will be made publicly available when the study is completed and published.<br><br>* typeset	
Additional data availability information:	

Powered by Editorial Manager® and ProduXion Manager® from Aries Systems Corporation

Manuscript

Click here to access/download;Manuscript;Sierpe - Manuscript.docx

Agenda-setting in the clinical encounter: A systematic review protocol

Author	Email	ORCiD	Affiliation
Ailyn Sierpe	ailyn.sierpe@dartmouth.edu	0000-0003-4369-9934	1
Renata W. Yen	renata.west.yen@dartmouth.edu	0000-0002-6856-7631	2, 1
Gabrielle Stevens	gabrielle.stevens@dartmouth.edu	0000-0001-9001-178X	1
Aricca D. Van Citters	aricca.d.van.citters@dartmouth.edu	0000-0002-2961-5661	1
Glyn Elwyn	glynelwyn@gmail.com	0000-0002-0917-6286	1
Catherine H. Saunders *[corresponding]*	catherine.hylas.saunders@dartmouth.edu	0000-0003-0819-6610	3, 1

No.	Affiliation
1	The Dartmouth Institute for Health Policy and Clinical Practice, Geisel School of Medicine at Dartmouth College, 1 M[illegible] Dr, Lebanon, NH, 03766, United States
2	Center for Technology and Behavioral Health, Geisel School of Medicine at Dartmouth College, 46 Centerra Pkwy, [illegible] 03766, United States
3	Dartmouth Health, 1 Medical Center Dr, Lebanon, NH, 03766, United States

Abstract

Introduction

Agenda-setting is a collaborative communication strategy used by a clinician before or at the start of a clinical encounter to work together with the patient to “elicit, propose, and organize” topics to be discussed during the encounter. While clinical visit agenda-setting has been acknowledged as an important element of patient-centered communication, the effectiveness of agenda-setting interventions in improving healthcare outcomes is unclear. To our knowledge, no systematic review has examined clinical visit agenda-setting interventions.

Methods and analysis

The primary aim of the systematic review will be to assess the effects of agenda-setting interventions on outcomes relating to the clinical encounter itself, patients, and clinicians, as well as any other study-specified outcomes. Our secondary aims will be to examine the characteristics and delivery attributes of agenda-setting interventions, as well as how agenda-setting has been operationalized and measured. We will search selected databases and gray literature from inception until March 2024. All studies comparing a clinical visit agenda-setting intervention with either usual care or another agenda-setting intervention will be included. Two independent reviewers will complete article screening and data extraction, with a third independent reviewer resolving any conflicts. We will assess all studies’ methodological quality and the quality of their evidence using standardized criteria. If a sufficient number of studies report the same outcomes, we will pool their results and perform a meta-analysis of those

outcomes. We will also synthesize all results qualitatively, regardless of whether we are able to complete a meta-analysis.

PROSPERO registration number

CRD42023468045

Introduction

Agenda-setting is a collaborative communication strategy used by clinicians before or at the start of a clinical encounter.[1,2] It has been defined as a practice in which the clinician works together with the patient to "elicit, propose, and organize" the topics to be discussed during the encounter.[1,3,4] Clinical visit agenda-setting is an important element of patient-centered communication,[5] and has been associated with positive outcomes such as greater patient satisfaction, increased likelihood of patients adhering to treatment recommendations, and clinicians' improved understanding of patient concerns.[1,3,6–8] Agenda-setting has also been shown to reduce the overall number of concerns that go unaddressed by the end of the visit, or that are not sufficiently resolved due to the patient raising them at the last minute.[1,9]

Although agenda-setting has been acknowledged as a valuable practice and is commonly included in medical education, many clinicians have a limited understanding of agenda-setting or may not perform it effectively, given the competing priorities and interests within encounters.[1,3,4,10–12] Naturalistic studies of clinical visit agenda-setting have also observed considerable variation in its real-world prevalence.[1,8,10,13] This is compounded by the challenge of measuring agenda-setting, which has been inconsistently defined and operationalized, without widespread adoption of any single standardized measuring tool.[1,4,14] For example, while some studies consider all instances of clinicians starting an encounter by asking an open-ended question to be agenda-setting,[15] others have placed specific time limits for when agenda-setting must occur, such as within the first five minutes.[13]

Studies of clinical visit agenda-setting have also adopted a wide range of approaches in designing interventions to increase or enhance agenda-setting. Namely, patient-facing interventions have largely centered on agenda-setting checklists or other tools to be completed by the patient or their care partner before or during the encounter, with some evidence of positive outcomes.[2,5,16–18] Meanwhile, clinician-facing interventions have typically involved training programs in agenda-setting skills, such as topic elicitation workshops.[3,16,18–22] Other studies have combined both of these approaches.[18]

Along with the diverse nature of the interventions themselves, the outcomes examined by these studies have also varied substantially. For instance, some have focused on the overall duration of the visit and the number of topics discussed,[5,23] or more specifically, the number of 'surprise' topics raised only at the end of the encounter.[1,18] As a broader measure of patient-centered communication, another study examined verbal activity, or the proportion of statements contributed by each speaker during the encounter.[5] Finally, other studies have used patient-reported measures of clinicians' communication skills and general satisfaction.[3,18] Such variation has contributed to the challenge of determining the overall effectiveness of existing clinical visit agenda-setting interventions.

Despite the relevance and value of clinical visit agenda-setting in patient-centered communication, there is currently a gap in our understanding of the best approaches to increasing the consistent adoption and quality of agenda-setting practices. We are also not aware of any previous comprehensive reviews of the evidence for clinical visit agenda-setting interventions. To address these gaps, our systematic review will primarily aim to assess the effects of agenda-setting interventions on outcomes relating to the clinical encounter itself, patients, and clinicians, as well as any other study-specified outcomes. Our secondary aims will be to examine the characteristics and delivery attributes of agenda-setting interventions, as well as how agenda-setting has been operationalized and measured.

Methods and analysis

We will conduct a systematic review of studies of clinical visit agenda-setting interventions. In response to our primary and secondary aims, we will address the following research questions:

1. What are the effects of clinical visit agenda-setting interventions on outcomes relating to the clinical encounter itself, patients, and clinicians, as well as any other study-specified outcomes?
2. What are the characteristics and delivery attributes of clinical visit agenda-setting interventions?
3. How has clinical visit agenda-setting been operationalized and measured?

We have registered this protocol with PROSPERO (CRD42023468045). We developed our methods with guidance from the Cochrane Handbook for Systematic Reviews of Interventions[24] and the Preferred Reporting Items for Systematic Review and Meta-Analysis Protocols (PRISMA-P) checklist (see Supporting Information 1).[25] After completing the review, we will report our methods and results using the full PRISMA guidelines.[26]

Preliminary searches

We conducted preliminary searches in PROSPERO, the Cochrane Database of Systematic Reviews, and Google Scholar to identify any existing systematic reviews and to assess the quantity of articles that could potentially be included. We used the keywords “agenda-setting,” “agenda-mapping,” “topic elicitation,” “topic solicitation,” “patient agenda,” “patient concerns,” “patient priorities,” “opening statement,” “visit opening,” and “visit structure.” We assessed the most cited literature reviews and randomized control trials identified from the searches. We did not find any systematic reviews of clinical visit agenda-setting.

Primary search strategy

We developed and piloted our search strategy (see Supporting Information 2) with the assistance of two biomedical research librarians at Dartmouth College. We will perform electronic searches in APA PsycInfo, Cochrane Central Register of Controlled Trials, Cochrane Database of Systematic Reviews, Cumulative Index to Nursing and Allied Health Literature, MEDLINE via PubMed, ProQuest, Scopus, and Web of Science from inception until March 2024 (see Table 1). As we did not identify any relevant MeSH terms referring to clinical visit agenda-setting, we will use sets of keywords instead. We will not use limits or language restrictions. A list of all searches performed will be included as a supplement to the final report.

Table 1. *Databases in primary search strategy*

Database name	Years covered
APA PsycInfo	1806–present
The Cochrane Library (Central Register of Controlled Trials)	1989–present
The Cochrane Library (Cochrane Database of Systematic Reviews)	1996–present
Cumulative Index to Nursing and Allied Health Literature	1981–present
MEDLINE via PubMed	1946–present
ProQuest	1861–present
Scopus	1788–present
Web of Science	1900–present

Secondary search strategy

We will supplement our primary search strategy with the following secondary search methods (see Supporting Information 2):

- Searching the reference lists of all included articles to find any articles not identified in our initial searches (backward citation search).
- Searching the citations of all included articles by using the 'cited by' feature in Web of Science (forward citation search).
- Searching Google Scholar and screening the first 25 pages of results. If results continue to be relevant after 25 pages, we will continue reviewing additional pages until two consecutive pages yield no relevant articles.
- Searching ClinicalTrials.gov for registered clinical trials.
- Searching key journals, relevant academic conference proceedings, and gray literature such as technical reports and works in progress.
- Searching reference lists provided by colleagues with expertise in research on clinical visit agenda-setting.

Inclusion criteria

Types of studies

We will include randomized studies of clinical visit agenda-setting interventions where a comparison is made to either no intervention (usual care) or to at least one other agenda-setting intervention (see Supporting Information 3). We will also include non-randomized studies, provided there is a comparison strategy (pre-post or quasi-experimental designs). To allow us to draw stronger conclusions on intervention effectiveness, we will exclude non-comparative designs and qualitative studies. As we anticipate there being relatively little research on this topic, based on our preliminary searches and knowledge of the literature in this field, we will include feasibility studies and pilot trials. We will also not impose restrictions on publication language. A colleague fluent in the target language will translate any non-English articles. Where this is not possible, we will use Google Translate and ask the authors to confirm the appropriateness of our interpretations.

Types of participants

We will include all participants involved in a clinical visit agenda-setting intervention, including patients, their care partners, clinicians, and any other groups. As agenda-setting can be practiced with any group or condition and in any clinical environment, we will include all clinical settings and participant groups.

Types of interventions

We will include all interventions that specifically aim to promote or increase clinical visit agenda-setting. The interventions may be patient-facing, clinician-facing, or both. We will exclude any interventions that only incidentally involve agenda-setting, such as those primarily focused on goal-setting. We have developed a working definition of agenda-setting for the purposes of this review by combining the characteristics of agenda-setting commonly described in the literature (see Supporting Information 3). We define agenda-setting as a practice in which a clinician works collaboratively with a patient to elicit, and often propose or organize, the topics to be discussed during a clinical encounter.[1,3] Some of the literature aiming to define and characterize agenda-setting has emphasized the need for it to be performed to exhaustion. This means the clinician continues to elicit additional topics until none remain.[2,4,14,27,28] We argue exhaustion is not a practical goal given the time limitations of clinical visits, so we instead focus on the general elicitation of topics. Additionally, while some researchers have understood agenda-setting as taking place at any time during a visit,[4,27] both observational studies and studies of agenda-setting interventions have acknowledged the positioning of agenda-setting before or early on in the encounter as best practice.[1,2,8,9,13,15,29] We will therefore only include interventions in which the agenda-setting occurs before or at the start of the encounter, rather than the end.

Types of outcomes

To address our primary aim, we will include all clinical and non-clinical outcomes relating to the clinical encounters themselves, patients, or clinicians. As we expect the literature on clinical visit agenda-setting will be limited, we will also include any other study-specified primary and secondary outcomes. Based on our preliminary searches, this may include but will not be limited to visit process outcomes, patient or care partner-reported outcomes, clinician-reported outcomes, and observer measures. To address our secondary aims, we will include outcomes relating to any agenda-setting measurement instruments, such as their reliability or validity.

Initial screening

We will upload all articles retrieved from the searches to Rayyan[30] or a similar systematic review management software. We will then remove any duplicate items. The data will be continuously backed up and managed by the software.

Two independent reviewers will first pilot our preliminary study inclusion criteria (see Supporting Information 3) with a small sample of articles. After confirming their shared understanding of the inclusion criteria and revising as needed, the reviewers will begin screening by reviewing the title and abstract of each article to determine whether it meets the final criteria. To maintain consistency, we will use a standardized list of study inclusion criteria with detailed descriptions of each point. Articles that pass this first round of screening will then advance onto full-text review. The two reviewers will again independently screen for articles matching the inclusion criteria. Any disagreements at either stage of screening will be resolved by discussion with a third independent reviewer.

Data extraction

We will extract data from the articles meeting our study inclusion criteria using a predesigned standardized data collection form. To extract information on intervention characteristics, we will include items adapted from the TIDieR (Template for Intervention Description and Replication) checklist in the data collection form.[31] We will extract details about the 1) authors, 2) publication year, 3) country where the study took place, 4) study design, 5) study aims and research questions, 6) participant characteristics and sample size, 7) study setting, 8) characteristics and duration of the intervention, 9) follow-up, 10) comparison conditions, 11) number of participants included in the analysis for both intervention and comparison groups, 12) measurement of clinical visit agenda-setting, and 13) all primary and secondary outcomes.

Two independent reviewers will first pilot the form using a small set of articles. After revising the form as needed, the reviewers will progress to full data extraction for the included studies, with disagreements again resolved by discussion with a third independent reviewer. We will attempt to obtain any missing data by contacting article authors. If we are unable to reach them, we will compute remaining values where appropriate.[32,33]

Methodological quality assessment

As our inclusion criteria cover multiple study designs, we will use two different risk-of-bias tools to evaluate the included studies: RoB 2 (the Cochrane risk-of-bias tool) for randomized control trials,[34] and ROBINS-I (Risk Of Bias In Non-randomised Studies - of Interventions) for non-randomized trials.[35] All reviewers will be trained on using the tools before beginning their assessments. Two independent reviewers will assign each study a rating of methodological quality using the risk-of-bias tool corresponding to its design. A third independent reviewer will resolve any disagreements. We will also use GRADE (Grading of Recommendations, Assessment, Development and Evaluation) to evaluate the certainty of the evidence reported.[36,37] We will use the RoB 2 and ROBINS-I results and other components of the included studies to apply the GRADE scoring.

Data synthesis

For any outcomes reported by at least three studies, we will pool the results and conduct a meta-analysis using statistical software. We will present dichotomous data as relative risks and continuous data as means or standardized mean differences, with all analyses using 95% confidence intervals. We will use a random effects model to account for population variability, assuming statistical significance at $p<0.05$. For quantitatively pooled results, we will assess heterogeneity using χ^2 and I^2 tests. For outcomes not included in the meta-analysis, we will qualitatively assess heterogeneity by visually inspecting the results of the individual studies to identify any notable outliers. If there is more than minimal heterogeneity ($p<0.10$ and $I^2>40\%$),[38,39] we will identify the sources of heterogeneity and assess their impact on the meta-analysis. We will assess reporting bias using a funnel plot, where effect estimates of the common outcome measure are plotted against trial sample size. For outcomes not included in the meta-analysis, we will assess reporting bias qualitatively.

We will also produce a narrative synthesis of the included studies.[40] The data from each study, including intervention characteristics, setting, participant characteristics, and enrollment metrics, will be used to build evidence tables according to the SWiM (Synthesis Without Meta-

analysis) reporting guidelines.[41] Using the evidence tables, we will qualitatively summarize the direction and intensity of the effects of the agenda-setting interventions. We will also summarize results relating to our secondary aims. To increase the validity of our narrative synthesis, we will adopt the participatory approach outlined in Practical Thematic Analysis[42] and will include a clinician and a patient partner as members of the research team. Our partners will take part in interpreting and summarizing the results of the included studies, as well as provide feedback on the initial results of the systematic review.

We will perform sensitivity analyses by removing the methodologically weakest studies, outliers in participant or intervention characteristics, and any studies where we computed missing values to determine whether the intervention effects change or remain the same. We will also perform subgroup analyses where there is sufficient data. While we will not predetermine subgroups, this would ideally include separate analyses of patient-facing interventions, clinician-facing interventions, and those combining both approaches.

Ethics and dissemination

The results of this systematic review will be submitted for publication in a peer-reviewed journal and presented at relevant conferences. We will report on the full review in accordance with the PRISMA (Preferred Reporting Items for Systematic Reviews and Meta-Analyses) statement.[26] In the event of any amendments to the protocol, the date of each amendment will be accompanied by a description of the change and its rationale. Changes will not be incorporated into the original protocol.

Discussion

Clinical visit agenda-setting has been repeatedly recognized as a best practice in patient-centered communication, with its ability to foster shared understanding and engagement between patients and clinicians in organizing and addressing topics of importance.[4,27] However, recent signals have pointed to substantial variation in how often agenda-setting actually occurs,[1,13] clinicians' ability to perform high-quality agenda-setting,[10,12] and the specific benefits of agenda-setting for different patient populations and clinical contexts.[1,3,9] Despite this, there has not yet been a comprehensive evaluation of interventions to increase or improve agenda-setting in the clinical encounter. To address this gap, this systematic review will evaluate the effectiveness of clinical visit agenda-setting interventions in improving outcomes for the encounters themselves, patients, and clinicians. Such a review has been long overdue, particularly given the broader value of agenda-setting as a path towards better aligning healthcare delivery with patients' individual needs.[6,7] By contributing to evidence on how to best promote clinical visit agenda-setting, we can inform ongoing efforts to develop, test, and implement effective targeted interventions that will serve to advance patient-centered communication as a whole.

Contributors

The protocol was developed by AS, with RWY, GS, GE, and CHS reviewing the research questions, search strategy, and terminology. AS wrote the first draft of the manuscript. RWY, GS, AVC, GE, and CHS contributed to writing and reviewing the manuscript. All authors have

reviewed and approved the final version of the manuscript. AS is the guarantor and CHS is the corresponding author.

Acknowledgements

We thank Heather Blunt and Elaina Vitale for their assistance in developing the search strategy.

Supporting Information captions

Supporting Information 1. PRISMA-P checklist.
Supporting Information 2. Piloted search strategy.
Supporting Information 3. Summary of inclusion criteria and agenda-setting definitions.

References

1. Hood-Medland EA, White AEC, Kravitz RL, Henry SG. Agenda setting and visit openings in primary care visits involving patients taking opioids for chronic pain. BMC Fam Pract. 2021;22: 4. doi:10.1186/s12875-020-01317-4

2. Robinson JD, Tate A, Heritage J. Agenda-setting revisited: When and how do primary-care physicians solicit patients' additional concerns? Patient Educ Couns. 2016;99: 718–723. doi:10.1016/j.pec.2015.12.009

3. Rodriguez HP, Anastario MP, Frankel RM, Odigie EG, Rogers WH, von Glahn T, et al. Can teaching agenda-setting skills to physicians improve clinical interaction quality? A controlled intervention. BMC Med Educ. 2008;8: 3. doi:10.1186/1472-6920-8-3

4. Gobat N, Kinnersley P, Gregory JW, Robling M. What is agenda setting in the clinical encounter? Consensus from literature review and expert consultation. Patient Educ Couns. 2015;98: 822–829. doi:10.1016/j.pec.2015.03.024

5. Wolff JL, Roter DL, Boyd CM, Roth DL, Echavarria DM, Aufill J, et al. Patient–Family Agenda Setting for Primary Care Patients with Cognitive Impairment: the SAME Page Trial. J Gen Intern Med. 2018;33: 1478–1486. doi:10.1007/s11606-018-4563-y

6. Safran DG, Taira DA, Rogers WH, Kosinski M, Ware JE, Tarlov AR. Linking primary care performance to outcomes of care. J Fam Pract. 1998;47: 213–220. Available: https://www.ncbi.nlm.nih.gov/pubmed/9752374

7. Stewart M, Brown JB, Donner A, McWhinney IR, Oates J, Weston WW, et al. The impact of patient-centered care on outcomes. J Fam Pract. 2000;49: 796–804. Available: https://www.ncbi.nlm.nih.gov/pubmed/11032203

8. Frankel RM, Salyers MP, Bonfils KA, Oles SK, Matthias MS. Agenda setting in psychiatric consultations: an exploratory study. Psychiatr Rehabil J. 2013;36: 195–201. doi:10.1037/prj0000004

9. Baker LH, O’connell D, Platt FW. “What else?” Setting the agenda for the clinical interview. Ann Intern Med. 2005;143: 766–770. doi:10.7326/0003-4819-143-10-200511150-00033

10. Roh H, Park KH, Jeon Y-J, Park SG, Lee J. Medical students’ agenda-setting abilities during medical interviews. Korean Journal of Medical Education. 2015. pp. 77–86. doi:10.3946/kjme.2015.27.2.77

11. Mishra MK, Saunders CH, Rodriguez HP, Shortell SM, Fisher E, Elwyn G. How do healthcare professionals working in accountable care organisations understand patient activation and engagement? Qualitative interviews across two time points. BMJ Open. 2018;8: e023068. doi:10.1136/bmjopen-2018-023068

12. Coyle AC, Yen RW, Elwyn G. Interrupted opening statements in clinical encounters: A scoping review. Patient Educ Couns. 2022;105: 2653–2663. doi:10.1016/j.pec.2022.03.026

13. Rey-Bellet S, Dubois J, Vannotti M, Zuercher M, Faouzi M, Devaud K, et al. Agenda Setting During Follow-Up Encounters in a University Primary Care Outpatient Clinic. Health Commun. 2017;32: 714–720. doi:10.1080/10410236.2016.1168003

14. Gobat N, Kinnersley P, Gregory JW, Pickles T, Hood K, Robling M. Measuring clinical skills in agenda-mapping (EAGL-I). Patient Educ Couns. 2015;98: 1214–1221. doi:10.1016/j.pec.2015.06.018

15. Singh Ospina N, Phillips KA, Rodriguez-Gutierrez R, Castaneda-Guarderas A, Gionfriddo MR, Branda ME, et al. Eliciting the Patient’s Agenda- Secondary Analysis of Recorded Clinical Encounters. J Gen Intern Med. 2019;34: 36–40. doi:10.1007/s11606-018-4540-5

16. Hoskins G, Williams B, Abhyankar P, Donnan P, Duncan E, Pinnock H, et al. Achieving Good Outcomes for Asthma Living (GOAL): mixed methods feasibility and pilot cluster randomised controlled trial of a practical intervention for eliciting, setting and achieving goals for adults with asthma. Trials. 2016;17: 584. doi:10.1186/s13063-016-1684-7

17. Middleton JF. Asking patients to write lists: feasibility study. BMJ. 1995;311: 34. doi:10.1136/bmj.311.6996.34

18. Middleton JF, McKinley RK, Gillies CL. Effect of patient completed agenda forms and doctors’ education about the agenda on the outcome of consultations: randomised controlled trial. BMJ. 2006;332: 1238–1242. doi:10.1136/bmj.38841.444861.7C

19. Mauksch LB, Hillenburg L, Robins L. The Establishing Focus protocol: Training for collaborative agenda setting and time management in the medical interview. Fam Syst Health. 2001;19: 147–157. doi:10.1037/h0089539

20. Brock DM, Mauksch LB, Witteborn S, Hummel J, Nagasawa P, Robins LS. Effectiveness of intensive physician training in upfront agenda setting. J Gen Intern Med. 2011;26: 1317–1323. doi:10.1007/s11606-011-1773-y

21. Haas LJ, Glazer K, Houchins J, Terry S. Improving the effectiveness of the medical visit: a brief visit-structuring workshop changes patients’ perceptions of primary care visits. Patient Educ Couns. 2006;62: 374–378. doi:10.1016/j.pec.2006.06.007

22. Stott NC, Rees M, Rollnick S, Pill RM, Hackett P. Professional responses to innovation in

clinical method: diabetes care and negotiating skills. Patient Educ Couns. 1996;29: 67–73. doi:10.1016/0738-3991(96)00935-4

23. Everden A, Early F, Homan K, Fuld J. P94 Patient Agenda Setting And Clinic Efficiency In Outpatients: An Individual Randomised Controlled Trial. Thorax. 2014;69: A118–A118. doi:10.1136/thoraxjnl-2014-206260.235

24. Higgins JPT, Thomas J, Chandler J, Cumpston M, Li T, Page MJ, et al. Cochrane Handbook for Systematic Reviews of Interventions. John Wiley & Sons; 2019. Available: https://play.google.com/store/books/details?id=cTqyDwAAQBAJ

25. Shamseer L, Moher D, Clarke M, Ghersi D, Liberati A, Petticrew M, et al. Preferred reporting items for systematic review and meta-analysis protocols (PRISMA-P) 2015: elaboration and explanation. BMJ. 2015;350: g7647. doi:10.1136/bmj.g7647

26. Page MJ, McKenzie JE, Bossuyt PM, Boutron I, Hoffmann TC, Mulrow CD, et al. The PRISMA 2020 statement: an updated guideline for reporting systematic reviews. BMJ. 2021;372: n71. doi:10.1136/bmj.n71

27. Gobat NH. Agenda setting in the clinical encounter: what is it, and is it measureable? phd, Cardiff University. 2014. Available: https://orca.cardiff.ac.uk/id/eprint/56395

28. Marvel MK, Epstein RM, Flowers K, Beckman HB. Soliciting the patient's agenda: have we improved? JAMA. 1999;281: 283–287. doi:10.1001/jama.281.3.283

29. Kowalski CP, McQuillan DB, Chawla N, Lyles C, Altschuler A, Uratsu CS, et al. "The Hand on the Doorknob": Visit Agenda Setting by Complex Patients and Their Primary Care Physicians. J Am Board Fam Med. 2018;31: 29–37. doi:10.3122/jabfm.2018.01.170167

30. Ouzzani M, Hammady H, Fedorowicz Z, Elmagarmid A. Rayyan—a web and mobile app for systematic reviews. Syst Rev. 2016;5: 210. doi:10.1186/s13643-016-0384-4

31. Hoffmann TC, Glasziou PP, Boutron I, Milne R, Perera R, Moher D, et al. Better reporting of interventions: template for intervention description and replication (TIDieR) checklist and guide. BMJ. 2014;348: g1687. doi:10.1136/bmj.g1687

32. Higgins JPT, Li T, Deeks JJ. Choosing effect measures and computing estimates of effect. Cochrane Handbook for Systematic Reviews of Interventions. Wiley; 2019. pp. 143–176. doi:10.1002/9781119536604.ch6

33. Rohatgi A. WebPlotDigitizer - Copyright 2010-2022 Ankit Rohatgi. [cited 1 Jun 2023]. Available: https://apps.automeris.io/wpd/

34. Sterne JAC, Savović J, Page MJ, Elbers RG, Blencowe NS, Boutron I, et al. RoB 2: a revised tool for assessing risk of bias in randomised trials. BMJ . 2019. p. l4898. doi:10.1136/bmj.l4898

35. Sterne JA, Hernán MA, Reeves BC, Savović J, Berkman ND, Viswanathan M, et al. ROBINS-I: a tool for assessing risk of bias in non-randomised studies of interventions. BMJ. 2016;355: i4919. doi:10.1136/bmj.i4919

36. Guyatt GH, Oxman AD, Schünemann HJ, Tugwell P, Knottnerus A. GRADE guidelines: a new series of articles in the Journal of Clinical Epidemiology. J Clin Epidemiol. 2011;64:

380–382. doi:10.1016/j.jclinepi.2010.09.011

37. GRADE handbook. [cited 1 Jun 2023]. Available: https://gdt.gradepro.org/app/handbook/handbook.html

38. Higgins JPT, Thompson SG. Quantifying heterogeneity in a meta-analysis. Stat Med. 2002;21: 1539–1558. doi:10.1002/sim.1186

39. Chapter 10: Analysing data and undertaking meta-analyses. [cited 2 Mar 2023]. Available: https://training.cochrane.org/handbook/current/chapter-10

40. Popay J, Roberts H, Sowden A, Petticrew M, Arai L, Rodgers M, et al. Guidance on the conduct of narrative synthesis in systematic reviews: A product from the ESRC Methods Programme. [object Object]: Lancaster University; 2006. doi:10.13140/2.1.1018.4643

41. Campbell M, McKenzie JE, Sowden A, Katikireddi SV, Brennan SE, Ellis S, et al. Synthesis without meta-analysis (SWiM) in systematic reviews: reporting guideline. BMJ. 2020;368: l6890. doi:10.1136/bmj.l6890

42. Saunders CH, Sierpe A, von Plessen C, Kennedy AM, Leviton LC, Bernstein SL, et al. Practical thematic analysis: a guide for multidisciplinary health services research teams engaging in qualitative analysis. BMJ. 2023;381: e074256. doi:10.1136/bmj-2022-074256

PRISMA-P Checklist

Click here to access/download

Supporting Information

Sierpe - S1.docx

Piloted Search Strategy

Click here to access/download

Supporting Information

Sierpe - S2.docx

Summary of Inclusion Criteria

Click here to access/download
Supporting Information
Sierpe - S3.docx
