# Supplementary material for: Agenda-setting in the clinical encounter: A systematic review protocol
Source: PLoS One. 2024 Oct 24;19(10):e0312613. doi: 10.1371/journal.pone.0312613 (PMC11500969; doi:10.1371/journal.pone.0312613)
Supplement: Attachment — Submitted filename: Response to Reviewers.docx [file pone.0312613.s005.docx]

## Reviewer #1

1. Have you planned to perform sensitivity analyses to explore the robustness of the findings?

Thank you for this suggestion. We have planned to perform sensitivity analyses, which we describe in **Data synthesis**, as below.

*We will perform sensitivity analyses by removing the methodologically weakest studies, outliers in participant or intervention characteristics, and any studies where we computed missing values to determine whether the intervention effects change or remain the same. We will also perform subgroup analyses, where there is sufficient data. While we will not predetermine specific subgroups, this would ideally include separate analyses of patient-facing interventions, clinician-facing interventions, and those combining both approaches. If the data is sufficient, we also aim to perform analyses by the included studies’ settings and participant characteristics.*

2. How you are going to pool the findings, it should be clearly described?

Thank you for your question. We have elaborated on our process of pooling study results in **Data synthesis**, as below.

*For any outcomes reported by at least two studies, we will pool the results and conduct a meta-analysis using statistical software.[[38,39]](https://paperpile.com/c/OdchLL/Em4e+AmJdT) We will present dichotomous data as relative risks and continuous data as means or standardized mean differences, with all analyses using 95% confidence intervals. As it is unlikely that the intervention effects will be identical, we will use a random effects model, assuming significance at p<0.05.[[40,41]](https://paperpile.com/c/OdchLL/rxFh+FpNQ) For quantitatively pooled results, we will assess heterogeneity using χ^2^ and I^2^ tests. For outcomes not included in the meta-analysis, we will qualitatively assess heterogeneity by visually inspecting the results of the individual studies to identify any notable outliers. If there is more than minimal heterogeneity (p<0.10 and I^2^>40%),[[39,42]](https://paperpile.com/c/OdchLL/gIoJW+AmJdT) we will identify the sources of heterogeneity and assess their impact on the meta-analysis. For outcomes reported by at least 10 studies, we will assess reporting bias using funnel plots, where effect estimates of the common outcome measure are plotted against trial sample size. We will then conduct Egger’s regressions to statistically assess for asymmetry, assuming significance at p<0.05. For outcomes reported by fewer than 10 studies, we will asses reporting bias qualitatively.[[43]](https://paperpile.com/c/OdchLL/LAfr)*

3. Are there enough articles to do systematic review and metaanalysis?

We appreciate your question, and the opportunity to clarify. We acknowledge the concern that there may be too few relevant articles to justify conducting a systematic review, as we are aware there is relatively little research on this topic. To ensure there is a sufficient quantity of articles ahead of conducting the systematic review, we performed preliminary searches with assistance from two biomedical research librarians. In the process of developing our search strategy, we piloted the database searches and noted the number of results, which we outlined in **Supporting Information 2. Piloted search strategy**. As these preliminary searches yielded over 1,000 articles (with deduplication across all databases searched), we are confident there are enough articles for a systematic review.

***Database****: Scopus*

***Dates covered****: 1788 to present*

***Date searched****: October 2023*

***Limits****: None used.*

***Search terms / results****: We searched titles, abstracts, and keywords in Scopus with the queries below. The search returned 1,023 results.*

***Table 8.*** *Scopus search queries and results*

| ***Search*** | ***Query*** | ***Results*** |
| --- | --- | --- |
| *1* | *TITLE-ABS-KEY ( "agenda setting" OR "set* agenda*" )* | *5,776* |
| *2* | *TITLE-ABS-KEY ( "agenda mapping" OR "map* agenda*" )* | *14* |
| *3* | *TITLE-ABS-KEY ( "agenda elicit*" OR "elicit* agenda*" )* | *7* |
| *4* | *TITLE-ABS-KEY ( "agenda solicit*" OR "solicit* agenda*" )* | *3* |
| *5* | *TITLE-ABS-KEY ( "topic setting" OR "set* topic*" )* | *261* |
| *6* | *TITLE-ABS-KEY ( "topic mapping" OR "map* topic*" )* | *157* |
| *7* | *TITLE-ABS-KEY ( "topic elicit*" OR "elicit* topic*" )* | *67* |
| *8* | *TITLE-ABS-KEY ( "topic solicit*" OR "solicit* topic*" )* | *8* |
| *9* | *TITLE-ABS-KEY ( "concern setting" OR "set* concern*" )* | *972* |
| *10* | *TITLE-ABS-KEY ( "concern mapping" OR "map* concern*" )* | *255* |
| *11* | *TITLE-ABS-KEY ( "concern elicit*" OR "elicit* concern*" )* | *222* |
| *12* | *TITLE-ABS-KEY ( "concern solicit*" OR "solicit* concern*" )* | *23* |
| *13* | *TITLE-ABS-KEY ( "priority elicit*" OR "elicit* priorit*" )* | *50* |
| *14* | *TITLE-ABS-KEY ( "priority solicit*" OR "solicit* priorit*" )* | *4* |
| *15* | *TITLE-ABS-KEY ( "patient* agenda*" )* | *145* |
| *16* | *TITLE-ABS-KEY ( "opening statement*" )* | *585* |
| *17* | *TITLE-ABS-KEY ( "visit opening*" OR "opening visit*" OR "encounter opening*" OR "opening encounter*" )* | *29* |
| *18* | *TITLE-ABS-KEY ( "visit structur*" OR "structur* visit*" OR "encounter structur*" OR "structur* encounter*" )* | *846* |
| *19* | *#1 OR #2 OR #3 OR #4 OR #5 OR #6 OR #7 OR #8 OR #9 OR #10 OR #11 OR #12 OR #13 OR #14 OR #15 OR #16 OR #17 OR #18* | *9,388* |
| *20* | *TITLE-ABS-KEY ( "patient*" OR "clinic*" )* | *15,942,003* |
| *21* | *TITLE-ABS-KEY ( "approach*" OR "brochure*" OR "checklist*" OR "coach*" OR "conversation analys*" OR "conversation* approach*" OR "dashboard*" OR "education*" OR "electronic health record*" OR "electronic medical record*" OR "form*" OR "framework*" OR "guide*" OR "guidance*" OR "handout*" OR "information*" OR "instrument*" OR "intervention*" OR "issue card*" OR "material*" OR "model*" OR "pamphlet*" OR "portal*" OR "prompt*" OR "protocol*" OR "question asking" OR "questionnaire*" OR "seminar*" OR "session*" OR "strateg*" OR "survey*" OR "technique*" OR "template*" OR "tool*" OR "training*" OR "webinar*" OR "workflow*" OR "worksheet*" OR "workshop*" )* | *50,838,378* |
| *22* | *#19 AND #20 AND #21* | *1,023* |

In our preliminary searches, we also identified multiple articles reporting the same outcomes. For instance, we found at least two articles each reporting on the occurrence of agenda-setting in the encounter, the number of patient concerns raised during the encounter, and the duration of the encounter. As we only need two articles reporting the same outcome to conduct a meta-analysis, we are also confident we will be able to do so. However, even if this were not the case, we would still be able to conduct a narrative synthesis of the included studies, as we discuss in **Data synthesis**.

*For any outcomes reported by at least two studies, we will pool the results and conduct a meta-analysis using statistical software.[[38,39]](https://paperpile.com/c/OdchLL/Em4e+AmJdT) We will present dichotomous data as relative risks and continuous data as means or standardized mean differences, with all analyses using 95% confidence intervals. As it is unlikely that the intervention effects will be identical, we will use a random effects model, assuming significance at p<0.05.[[40,41]](https://paperpile.com/c/OdchLL/rxFh+FpNQ) For quantitatively pooled results, we will assess heterogeneity using χ^2^ and I^2^ tests. For outcomes not included in the meta-analysis, we will qualitatively assess heterogeneity by visually inspecting the results of the individual studies to identify any notable outliers. If there is more than minimal heterogeneity (p<0.10 and I^2^>40%),[[39,42]](https://paperpile.com/c/OdchLL/gIoJW+AmJdT) we will identify the sources of heterogeneity and assess their impact on the meta-analysis. For outcomes reported by at least 10 studies, we will assess reporting bias using funnel plots, where effect estimates of the common outcome measure are plotted against trial sample size. We will then conduct Egger’s regressions to statistically assess for asymmetry, assuming significance at p<0.05. For outcomes reported by fewer than 10 studies, we will asses reporting bias qualitatively.[[43]](https://paperpile.com/c/OdchLL/LAfr)*

*We will also produce a narrative synthesis of the included studies.[[44]](https://paperpile.com/c/OdchLL/MO7pT) The data from each study, including intervention characteristics, setting, participant characteristics, and enrollment metrics, will be used to build evidence tables according to the SWiM (Synthesis Without Meta-analysis) reporting guidelines.[[45]](https://paperpile.com/c/OdchLL/9BzOL) Using the evidence tables, we will qualitatively summarize the direction and intensity of the effects of the agenda-setting interventions. We will also summarize results relating to our secondary aims. To increase the validity of our narrative synthesis, we will adopt the participatory approach outlined in Practical Thematic Analysis[[46]](https://paperpile.com/c/OdchLL/w4weq) and will include a clinician and a patient partner as members of the research team. Our partners will take part in interpreting and summarizing the results of the included studies, as well as provide feedback on the initial results of the systematic review.*

*We will perform sensitivity analyses by removing the methodologically weakest studies, outliers in participant or intervention characteristics, and any studies where we computed missing values to determine whether the intervention effects change or remain the same. We will also perform subgroup analyses, where there is sufficient data. While we will not predetermine specific subgroups, this would ideally include separate analyses of patient-facing interventions, clinician-facing interventions, and those combining both approaches. If the data is sufficient, we also aim to perform analyses by the included studies’ settings and participant characteristics.*

4. Study period needs justification, as you planned to start from 1788?

Thank you for this suggestion. We have updated our **Primary search strategy** to justify our search dates.

*We developed and piloted our search strategy (see Supporting Information 2) with the assistance of two biomedical research librarians at Dartmouth College. We will perform electronic searches in APA PsycInfo, Cochrane Central Register of Controlled Trials, Cochrane Database of Systematic Reviews, Cumulative Index to Nursing and Allied Health Literature, MEDLINE via PubMed, ProQuest, Scopus, and Web of Science. With the broad nature of clinical visit agenda-setting, it is difficult to determine a specific start date for when interventions would have first been developed. We will therefore search for articles from database inception until date of search (see Table 1). As we did not identify any relevant MeSH terms referring to clinical visit agenda-setting, we will use sets of keywords instead. We will not use limits or language restrictions. A list of all searches performed will be included as a supplement to the final report.*

5. How you are going to determine sufficient number of studies report the same outcomes, to pool their results?

Thank you for your question. We have also touched on this in our response to your earlier question (3), regarding how we know we have a sufficient number of articles to complete a meta-analysis. Namely, we have already identified studies from our preliminary searches conducted while developing our search strategy that report the same outcomes and would therefore be able to be pooled quantitatively.

In a practical sense, we will be able to organize studies reporting on the same outcomes in our data extraction process, which we have detailed below in **Data extraction**. Our standardized data collection form allows us to group multiple studies reporting on the same outcomes, as these studies will share columns in the spreadsheet. We will therefore be able to easily monitor which studies share which of the same outcomes throughout data extraction.

*We will extract data from the articles meeting our study inclusion criteria using a predesigned standardized data collection form. The form will consist of a spreadsheet, with the rows listing the articles and the columns indicating the data points to be extracted. To extract information on intervention characteristics, we will include items adapted from the TIDieR (Template for Intervention Description and Replication) checklist in the data collection form.[[31]](https://paperpile.com/c/OdchLL/j5RT6) We will extract details about the 1) authors, 2) publication year, 3) country where the study took place, 4) study design, 5) study aims and research questions, 6) participant characteristics and sample size, 7) study setting, 8) characteristics and duration of the intervention, 9) follow-up, 10) comparison conditions, 11) number of participants included in the analysis for both intervention and comparison groups, 12) measurement of clinical visit agenda-setting, and 13) all primary and secondary outcomes.*

*Two independent reviewers will first pilot the form using a small set of articles. After revising the form as needed, the reviewers will progress to full data extraction for the included studies, with disagreements again resolved by discussion with a third independent reviewer. We will attempt to obtain any missing data by contacting article authors. If we are unable to reach them, we will compute remaining values where appropriate.[[32,33]](https://paperpile.com/c/OdchLL/2cxgR+6sZ0K)*

6. The review question should precise and overall writing issues should be revised

Thank you for your comment. We acknowledge that our research questions have been written more broadly than those in some other systematic review protocols. This is intentional. Our team has been working on a program of research surrounding clinical visit agenda-setting and has seen that interventions in this area are highly diverse in their designs, target populations, and outcomes of interest. We aim to highlight this range in our **Introduction** below. As this will be the first systematic review examining clinical visit agenda-setting interventions that we know of, we wrote our research questions with the aim of capturing the full diversity of studies of these interventions.

*Although agenda-setting has been acknowledged as a valuable practice and is commonly included in medical education, many clinicians have a limited understanding of agenda-setting or may not perform it effectively, given the competing priorities and interests within encounters.[[1,3,4,10–12]](https://paperpile.com/c/OdchLL/andbN+K5gjH+dr1kK+peVqU+Rjsql+o2yZL) Naturalistic studies of clinical visit agenda-setting have also observed considerable variation in its real-world prevalence.[[1,8,10,13]](https://paperpile.com/c/OdchLL/peVqU+0cKyH+RoIch+K5gjH) This is compounded by the challenge of measuring agenda-setting, which has been inconsistently defined and operationalized, without widespread adoption of any single standardized measuring tool.[[1,4,14]](https://paperpile.com/c/OdchLL/peVqU+dr1kK+on8Du) For example, while some studies consider all instances of clinicians starting an encounter by asking an open-ended question to be agenda-setting,[[15]](https://paperpile.com/c/OdchLL/UCg3j) others have placed specific time limits for when agenda-setting must occur, such as within the first five minutes.[[13]](https://paperpile.com/c/OdchLL/0cKyH)*

*Studies of clinical visit agenda-setting have also adopted a wide range of approaches in designing interventions to increase or enhance agenda-setting. Namely, patient-facing interventions have largely centered on agenda-setting checklists or other tools to be completed by the patient or their care partner before or during the encounter, with some evidence of positive outcomes.[[2,5,16–18]](https://paperpile.com/c/OdchLL/J0k5b+zmX1i+0cx64+ywJsi+fzNhB) Meanwhile, clinician-facing interventions have typically involved training programs in agenda-setting skills, such as topic elicitation workshops.[[3,16,18–22]](https://paperpile.com/c/OdchLL/ywJsi+r4xce+v3XVK+andbN+Rtd8N+zmX1i+YBZPA) Other studies have combined both of these approaches.[[18]](https://paperpile.com/c/OdchLL/ywJsi)*

*Along with the diverse nature of the interventions themselves, the outcomes examined by these studies have also varied substantially. For instance, some have focused on the overall duration of the visit and the number of topics discussed,[[5,23]](https://paperpile.com/c/OdchLL/J0k5b+uLunQ) or more specifically, the number of ‘surprise’ topics raised only at the end of the encounter.[[1,18]](https://paperpile.com/c/OdchLL/peVqU+ywJsi) As a broader measure of patient-centered communication, another study examined verbal activity, or the proportion of statements contributed by each speaker during the encounter.[[5]](https://paperpile.com/c/OdchLL/J0k5b) Finally, other studies have used patient-reported measures of clinicians’ communication skills and general satisfaction.[[3,18]](https://paperpile.com/c/OdchLL/andbN+ywJsi) Such variation has contributed to the challenge of determining the overall effectiveness of existing clinical visit agenda-setting interventions.*

With regards to the writing, we have aimed to address any errors with close revision of the manuscript by multiple native US English-speaking colleagues, as the lead author is a native English speaker but was trained in the United Kingdom.

7. Better to provide a thorough and detailed explanation of the interventions or exposures being examined, including precise descriptions that would enable others to replicate the study or determine its relevance to different contexts.

Thank you for your suggestion. We have highlighted the nature of the interventions being examined in our **Introduction** below, with specific examples from the literature.

*Studies of clinical visit agenda-setting have also adopted a wide range of approaches in designing interventions to increase or enhance agenda-setting. Namely, patient-facing interventions have largely centered on agenda-setting checklists or other tools to be completed by the patient or their care partner before or during the encounter, with some evidence of positive outcomes.[[2,5,16–18]](https://paperpile.com/c/OdchLL/J0k5b+zmX1i+0cx64+ywJsi+fzNhB) Meanwhile, clinician-facing interventions have typically involved training programs in agenda-setting skills, such as topic elicitation workshops.[[3,16,18–22]](https://paperpile.com/c/OdchLL/ywJsi+r4xce+v3XVK+andbN+Rtd8N+zmX1i+YBZPA) Other studies have combined both of these approaches.[[18]](https://paperpile.com/c/OdchLL/ywJsi)*

To enable replication and ensure clarity in the specific interventions being examined, we have included our precise definition of clinical visit agenda-setting in **Types of interventions** and in **Supporting Information 3. Summary of inclusion criteria and agenda-setting definitions**, with the latter showing how our team arrived at this definition based on other researchers’ conceptualizations of agenda-setting in the literature.

*We will include all interventions that specifically aim to promote or increase clinical visit agenda-setting. The interventions may be patient-facing, clinician-facing, or both. We will exclude any interventions that only incidentally involve agenda-setting, such as those primarily focused on goal-setting. We have developed a working definition of agenda-setting for the purposes of this review by combining the characteristics of agenda-setting commonly described in the literature (see Supporting Information 3). We define agenda-setting as a practice in which a clinician works collaboratively with a patient to elicit, and often propose or organize, the topics to be discussed during a clinical encounter.[[1,3]](https://paperpile.com/c/OdchLL/peVqU+andbN) Some of the literature aiming to define and characterize agenda-setting has emphasized the need for it to be performed to exhaustion. This means the clinician continues to elicit additional topics until none remain.[[2,4,14,27,28]](https://paperpile.com/c/OdchLL/on8Du+dr1kK+LsJkr+hmZPU+fzNhB) We argue exhaustion is not a practical goal given the time limitations of clinical visits, so we instead focus on the general elicitation of topics. We acknowledge that exhaustion may be appropriate for assessing the quality of agenda-setting, i.e. scoring agenda-setting quality in encounters.[[14]](https://paperpile.com/c/OdchLL/on8Du) Additionally, while some researchers have understood agenda-setting as taking place at any time during a visit,[[4,27]](https://paperpile.com/c/OdchLL/dr1kK+LsJkr) both observational studies and studies of agenda-setting interventions have acknowledged the positioning of agenda-setting before or early on in the encounter as best practice.[[1,2,8,9,13,15,29]](https://paperpile.com/c/OdchLL/fzNhB+0cKyH+peVqU+jYwZ9+RoIch+UCg3j+PNIBz) We will therefore only include interventions in which the agenda-setting occurs before or at the start of the encounter, rather than the end.*

***Table 1.*** *Summary of preliminary study inclusion criteria*

| ***Order*** | ***Category*** | ***Inclusion criterion*** |
| --- | --- | --- |
| *1* | *Study design* | *Include all randomized trials comparing an agenda-setting intervention to either no intervention (usual care) or to at least one other agenda-setting intervention. Include non-randomized trials only if there is also a comparison (pre-post or quasi-experimental designs). Include feasibility studies and pilot trials. Exclude non-comparative designs and qualitative studies.* |
| *2* | *Population* | *Include all participants and clinical settings.* |
| *3* | *Intervention* | *Include all interventions specifically aiming to promote or increase clinical visit agenda-setting. Exclude interventions only incidentally involving agenda-setting.* ***Agenda-setting is a practice in which a clinician works collaboratively with a patient to elicit, and often propose or organize, the topics to be discussed during a clinical encounter.***[*[14,20,22,34,36,37]*](https://paperpile.com/c/EfSBlp/WCfT2+FArY2+BDpsd+rQcAL+z1Z8x+m17RC) ***It must occur before or at the start of the encounter, but does not need to be performed to exhaustion.***[*[15,20,26,37]*](https://paperpile.com/c/EfSBlp/CjJZf+yKlie+FArY2+rQcAL) |
| *4* | *Outcomes* | *Include all study-specified primary and secondary outcomes. Include all units of measurement used in these outcomes.* |

***Table 2.*** *Development of our working definition of clinical visit agenda-setting based on existing definitions in the literature*

| ***Definition of agenda-setting*** | ***Comment*** |
| --- | --- |
| *“Setting an agenda (i.e. a set of discussion topics of importance to the patient or the clinician to be covered in the encounter)” with a patient”*[*[36]*](https://paperpile.com/c/EfSBlp/WCfT2) | *Topics may be important to* ***either the patient or clinician*** |
| *“Agenda-mapping is a collaborative communication strategy that enables clinicians and patients to establish shared focus on what to talk about by mapping options and agreeing priorities”*[*[20]*](https://paperpile.com/c/EfSBlp/FArY2) | *Covers topic* ***organization****, emphasizes equal* ***collaboration*** |
| *“Patients talk about their concerns, wishes, requests, and/or goals, clinicians raise subjects they consider important, [...] clinicians and patients agree shared priorities, a focus of what to talk about during the session is agreed, the conversation is collaborative, patients are involved and engaged in the conversation, a broad overview of potential discussion topics is constructed, the rest of of the consultation is structured based on the shared agenda”*[*[14]*](https://paperpile.com/c/EfSBlp/BDpsd) | *Topics are raised by* ***either the patient or clinician****, engaging in* ***collaborative identification*** *and* ***prioritization*** *of topics* |
| *“To what extent did the clinician attempt to identify all possible talk topics upfront [...] and prioritize and agree a shared focus?”*[*[20,37]*](https://paperpile.com/c/EfSBlp/FArY2+rQcAL) | *Items are from a validated measure of agenda-mapping (EAGL-I), covers topic* ***elicitation*** *and* ***organization****, must be* ***collaborative****, and occur at the* ***start of the encounter*** |
| *“A communication strategy physicians use at the beginning of clinical visits to elicit, propose, and organize a complete list of topics to be covered”*[*[15]*](https://paperpile.com/c/EfSBlp/CjJZf) | *Covers topic* ***elicitation*** *and* ***organization****, must occur at the* ***start of the encounter*** |
| *“The ability of patients and their physicians to work together to identify and align priorities for a visit”*[*[34]*](https://paperpile.com/c/EfSBlp/z1Z8x) | ***Identification*** *and* ***prioritization*** *of topics,* ***collaborative*** |
| *“Soliciting patients’ chief complaints with open-ended question formats such as “What can I do for you?” or “How can I help?” [...] This type of agenda pursuit has its greatest effects when performed ‘early’ or ‘up front’ during visits”*[*[26]*](https://paperpile.com/c/EfSBlp/yKlie) | *Emphasizes* ***soliciting*** *topics, should occur at the* ***start of the encounter*** |
| *“Agenda setting is a strategy that seeks to improve the effectiveness of patient-clinician interactions by establishing relational “ground rules,” identifying priorities, and negotiating conversational focus”*[*[22]*](https://paperpile.com/c/EfSBlp/m17RC) | ***Identification*** *and* ***organization*** *of topics, establishing* ***patient-clinician relationship*** |

As part of our data extraction process, we will extract details on the characteristics of the interventions from the studies included in our review. These characteristics will be summarized and presented in a table as part of our results manuscript, which we hope will contribute to other researchers’ ability to replicate the results and determine the specific relevance of these interventions.

*To extract information on intervention characteristics, we will include items adapted from the TIDieR (Template for Intervention Description and Replication) checklist in the data collection form.[[31]](https://paperpile.com/c/OdchLL/j5RT6) We will extract details about the 1) authors, 2) publication year, 3) country where the study took place, 4) study design, 5) study aims and research questions, 6) participant characteristics and sample size, 7) study setting, 8) characteristics and duration of the intervention, 9) follow-up, 10) comparison conditions, 11) number of participants included in the analysis for both intervention and comparison groups, 12) measurement of clinical visit agenda-setting, and 13) all primary and secondary outcomes.*

8. Specific techniques to synthesize the data should be precisely described?

We appreciate your suggestion to elaborate on our data synthesis process. In response to comments from both reviewers, we have described our methods and their rationale in greater detail in **Data synthesis**, as below.

*For any outcomes reported by at least two studies, we will pool the results and conduct a meta-analysis using statistical software.[[38,39]](https://paperpile.com/c/OdchLL/Em4e+AmJdT) We will present dichotomous data as relative risks and continuous data as means or standardized mean differences, with all analyses using 95% confidence intervals. As it is unlikely that the intervention effects will be identical, we will use a random effects model, assuming significance at p<0.05.[[40,41]](https://paperpile.com/c/OdchLL/rxFh+FpNQ) For quantitatively pooled results, we will assess heterogeneity using χ^2^ and I^2^ tests. For outcomes not included in the meta-analysis, we will qualitatively assess heterogeneity by visually inspecting the results of the individual studies to identify any notable outliers. If there is more than minimal heterogeneity (p<0.10 and I^2^>40%),[[39,42]](https://paperpile.com/c/OdchLL/gIoJW+AmJdT) we will identify the sources of heterogeneity and assess their impact on the meta-analysis. For outcomes reported by at least 10 studies, we will assess reporting bias using funnel plots, where effect estimates of the common outcome measure are plotted against trial sample size. We will then conduct Egger’s regressions to statistically assess for asymmetry, assuming significance at p<0.05. For outcomes reported by fewer than 10 studies, we will asses reporting bias qualitatively.[[43]](https://paperpile.com/c/OdchLL/LAfr)*

*We will also produce a narrative synthesis of the included studies.[[44]](https://paperpile.com/c/OdchLL/MO7pT) The data from each study, including intervention characteristics, setting, participant characteristics, and enrollment metrics, will be used to build evidence tables according to the SWiM (Synthesis Without Meta-analysis) reporting guidelines.[[45]](https://paperpile.com/c/OdchLL/9BzOL) Using the evidence tables, we will qualitatively summarize the direction and intensity of the effects of the agenda-setting interventions. We will also summarize results relating to our secondary aims. To increase the validity of our narrative synthesis, we will adopt the participatory approach outlined in Practical Thematic Analysis[[46]](https://paperpile.com/c/OdchLL/w4weq) and will include a clinician and a patient partner as members of the research team. Our partners will take part in interpreting and summarizing the results of the included studies, as well as provide feedback on the initial results of the systematic review.*

*We will perform sensitivity analyses by removing the methodologically weakest studies, outliers in participant or intervention characteristics, and any studies where we computed missing values to determine whether the intervention effects change or remain the same. We will also perform subgroup analyses, where there is sufficient data. While we will not predetermine specific subgroups, this would ideally include separate analyses of patient-facing interventions, clinician-facing interventions, and those combining both approaches. If the data is sufficient, we also aim to perform analyses by the included studies’ settings and participant characteristics.*

9. What is standardized data collection form, how, it should be clear.

Thank you for your question. In response, we have added further detail in describing the data collection form in **Data extraction**.

*We will extract data from the articles meeting our study inclusion criteria using a predesigned standardized data collection form. The form will consist of a spreadsheet, with the rows listing the articles and the columns indicating the data points to be extracted. To extract information on intervention characteristics, we will include items adapted from the TIDieR (Template for Intervention Description and Replication) checklist in the data collection form.[[31]](https://paperpile.com/c/OdchLL/j5RT6) We will extract details about the 1) authors, 2) publication year, 3) country where the study took place, 4) study design, 5) study aims and research questions, 6) participant characteristics and sample size, 7) study setting, 8) characteristics and duration of the intervention, 9) follow-up, 10) comparison conditions, 11) number of participants included in the analysis for both intervention and comparison groups, 12) measurement of clinical visit agenda-setting, and 13) all primary and secondary outcomes.*

## Reviewer #2

The comment is provided in track change. You just need to refine those like: The list number of studies to be considered to conduct meta analysis. The planned sensitivity and subgroup analysis is limited so broaden to setting, and population group.

Thank you very much for your comments as provided in tracked changes. We have endeavored to address these comments in full below, and hope our responses are to your satisfaction.

We have specifically responded to your comments on the number of studies needed to conduct a meta-analysis and our planned sensitivity and subgroup analyses under points (5) and (8) below, respectively.

1. Why not beyound this time?

Thank you for this question. We had previously specified the month at the time of our initial manuscript submission. We have updated this to reflect that articles from inception up until the time we conduct the search will be included in our screening.

*We will search selected databases (APA PsycInfo, Cochrane Central Register of Controlled Trials, Cochrane Database of Systematic Reviews, Cumulative Index to Nursing and Allied Health Literature, MEDLINE via PubMed, ProQuest, Scopus, and Web of Science) and gray literature from inception until date of search.*

*We will perform electronic searches in APA PsycInfo, Cochrane Central Register of Controlled Trials, Cochrane Database of Systematic Reviews, Cumulative Index to Nursing and Allied Health Literature, MEDLINE via PubMed, ProQuest, Scopus, and Web of Science. With the broad nature of clinical visit agenda-setting, it is difficult to determine a specific start date for when interventions would have first been developed. We will therefore search for articles from database inception until date of search (see Table 1).*

2. Specify the selected databases

Thank you for your suggestion. We had previously only specified the selected databases in the **Primary search strategy** (see **Table 1**). We have now updated the **Abstract** to list the databases as well.

*We will search selected databases (APA PsycInfo, Cochrane Central Register of Controlled Trials, Cochrane Database of Systematic Reviews, Cumulative Index to Nursing and Allied Health Literature, MEDLINE via PubMed, ProQuest, Scopus, and Web of Science) and gray literature from inception until date of search.*

3. Discuss about the the primary outcome measurements. Which unit of measurement will be taken or which one not to be taken and justify why?

Thank you for raising this point. In response, we have clarified our approach to outcomes’ units of measurement in both **Types of outcomes** and **Supporting Information 3. Summary of inclusion criteria and agenda-setting definitions**.

*To address our primary aim, we will include all clinical and non-clinical outcomes relating to the clinical encounters themselves, patients, or clinicians. As our search strategy piloting indicated a relatively small volume of literature (see Supporting Information 2), we will also include any other study-specified primary and secondary outcomes. Based on our preliminary searches, this may include but will not be limited to visit process outcomes, patient or care partner-reported outcomes, clinician-reported outcomes, and observer measures. To address our secondary aims, we will include outcomes relating to any agenda-setting measurement instruments, such as their reliability or validity. With the highly diverse nature of our outcomes of interest, we will include all units of measurements used in the included studies.*

Outcomes: *Include all units of measurement used in these outcomes.*

4. How small is small?

Thank you for your question. In response, we have specified the number of articles needed in piloting our study inclusion criteria.

*Two independent reviewers will first pilot our preliminary study inclusion criteria (see Supporting Information 3) with approximately 50-100 article abstracts. After confirming their shared understanding of the inclusion criteria and revising as needed, the reviewers will begin screening by reviewing the title and abstract of each article to determine whether it meets the final criteria. To maintain consistency, we will use a standardized list of study inclusion criteria with detailed descriptions of each point. Articles that pass this first round of screening will then advance onto full-text review. The two reviewers will again independently screen for articles matching the inclusion criteria. Any disagreements at either stage of screening will be resolved by discussion with a third independent reviewer.*

5. What if it is two studies, if they are homogeneous still you can pool, please justify the lower limit of three studies

Many thanks for your suggestion. We have decreased the threshold for meta-analysis to two studies, as recommended by Cochrane.

*For any outcomes reported by at least two studies, we will pool the results and conduct a meta-analysis using statistical software.[[38,39]](https://paperpile.com/c/OdchLL/Em4e+AmJdT)*

6. What if you couldn't do random effect model because of the number of studies you found? let me say below five. you better consider the fixed effect model in that case.

Thank you for this suggestion on our choice of model, which we understand is a popular subject of debate. Given the diverse nature of the interventions likely to be included in our meta-analysis, we are assuming that the intervention effects are unlikely to be truly identical. Following the recommendations from Cochrane, we have chosen to use a random-effects model to match this assumption.[[39]](https://paperpile.com/c/EfSBlp/SuAg) We have added a justification for our choice in **Data synthesis**, as below.

We note that we will conduct a narrative analysis of all studies included in our review, regardless of their inclusion in our formal meta-analysis. We will therefore still be able to report on results for any outcomes reported by too few studies to allow for a random-effects model.

*For any outcomes reported by at least two studies, we will pool the results and conduct a meta-analysis using statistical software.[[38,39]](https://paperpile.com/c/OdchLL/Em4e+AmJdT) We will present dichotomous data as relative risks and continuous data as means or standardized mean differences, with all analyses using 95% confidence intervals. As it is unlikely that the intervention effects will be identical, we will use a random effects model, assuming significance at p<0.05.[[40,41]](https://paperpile.com/c/OdchLL/rxFh+FpNQ) For quantitatively pooled results, we will assess heterogeneity using χ^2^ and I^2^ tests. For outcomes not included in the meta-analysis, we will qualitatively assess heterogeneity by visually inspecting the results of the individual studies to identify any notable outliers. If there is more than minimal heterogeneity (p<0.10 and I^2^>40%),[[39,42]](https://paperpile.com/c/OdchLL/gIoJW+AmJdT) we will identify the sources of heterogeneity and assess their impact on the meta-analysis. For outcomes reported by at least 10 studies, we will assess reporting bias using funnel plots, where effect estimates of the common outcome measure are plotted against trial sample size. We will then conduct Egger’s regressions to statistically assess for asymmetry, assuming significance at p<0.05. For outcomes reported by fewer than 10 studies, we will asses reporting bias qualitatively.[[43]](https://paperpile.com/c/OdchLL/LAfr)*

7. The number of included studies matter for you to assess reporting bias, discuss this here on the protocol.

Thank you for this suggestion. We have specified the minimum number of studies needed to assess reporting bias quantitatively.

*For outcomes reported by at least 10 studies, we will assess reporting bias using funnel plots, where effect estimates of the common outcome measure are plotted against trial sample size. We will then conduct Egger’s regressions to statistically assess for asymmetry, assuming significance at p<0.05. For outcomes reported by fewer than 10 studies, we will asses reporting bias qualitatively.[[43]](https://paperpile.com/c/OdchLL/LAfr)*

8. Consider study setting and sociodemographic characteristics for sensitivity analysis as this may affect the outcome

Thank you for your excellent suggestion. In response, we have expanded our planned analyses as below.

*We will perform sensitivity analyses by removing the methodologically weakest studies, outliers in participant or intervention characteristics, and any studies where we computed missing values to determine whether the intervention effects change or remain the same. We will also perform subgroup analyses, where there is sufficient data. While we will not predetermine specific subgroups, this would ideally include separate analyses of patient-facing interventions, clinician-facing interventions, and those combining both approaches. If the data is sufficient, we also aim to perform analyses by the included studies’ settings and participant characteristics.*

## References

1. [Hood-Medland EA, White AEC, Kravitz RL, Henry SG. Agenda setting and visit openings in primary care visits involving patients taking opioids for chronic pain. BMC Fam Pract. 2021;22: 4. doi:](http://paperpile.com/b/OdchLL/peVqU)[10.1186/s12875-020-01317-4](http://dx.doi.org/10.1186/s12875-020-01317-4)
2. [Robinson JD, Tate A, Heritage J. Agenda-setting revisited: When and how do primary-care physicians solicit patients’ additional concerns? Patient Educ Couns. 2016;99: 718–723. doi:](http://paperpile.com/b/OdchLL/fzNhB)[10.1016/j.pec.2015.12.009](http://dx.doi.org/10.1016/j.pec.2015.12.009)
3. [Rodriguez HP, Anastario MP, Frankel RM, Odigie EG, Rogers WH, von Glahn T, et al. Can teaching agenda-setting skills to physicians improve clinical interaction quality? A controlled intervention. BMC Med Educ. 2008;8: 3. doi:](http://paperpile.com/b/OdchLL/andbN)[10.1186/1472-6920-8-3](http://dx.doi.org/10.1186/1472-6920-8-3)
4. [Gobat N, Kinnersley P, Gregory JW, Robling M. What is agenda setting in the clinical encounter? Consensus from literature review and expert consultation. Patient Educ Couns. 2015;98: 822–829. doi:](http://paperpile.com/b/OdchLL/dr1kK)[10.1016/j.pec.2015.03.024](http://dx.doi.org/10.1016/j.pec.2015.03.024)
5. [Wolff JL, Roter DL, Boyd CM, Roth DL, Echavarria DM, Aufill J, et al. Patient–Family Agenda Setting for Primary Care Patients with Cognitive Impairment: the SAME Page Trial. J Gen Intern Med. 2018;33: 1478–1486. doi:](http://paperpile.com/b/OdchLL/J0k5b)[10.1007/s11606-018-4563-y](http://dx.doi.org/10.1007/s11606-018-4563-y)
6. [Safran DG, Taira DA, Rogers WH, Kosinski M, Ware JE, Tarlov AR. Linking primary care performance to outcomes of care. J Fam Pract. 1998;47: 213–220. Available:](http://paperpile.com/b/OdchLL/kxE0S) <https://www.ncbi.nlm.nih.gov/pubmed/9752374>
7. [Stewart M, Brown JB, Donner A, McWhinney IR, Oates J, Weston WW, et al. The impact of patient-centered care on outcomes. J Fam Pract. 2000;49: 796–804. Available:](http://paperpile.com/b/OdchLL/mPkT8) <https://www.ncbi.nlm.nih.gov/pubmed/11032203>
8. [Frankel RM, Salyers MP, Bonfils KA, Oles SK, Matthias MS. Agenda setting in psychiatric consultations: an exploratory study. Psychiatr Rehabil J. 2013;36: 195–201. doi:](http://paperpile.com/b/OdchLL/RoIch)[10.1037/prj0000004](http://dx.doi.org/10.1037/prj0000004)
9. [Baker LH, O’connell D, Platt FW. “What else?” Setting the agenda for the clinical interview. Ann Intern Med. 2005;143: 766–770. doi:](http://paperpile.com/b/OdchLL/PNIBz)[10.7326/0003-4819-143-10-200511150-00033](http://dx.doi.org/10.7326/0003-4819-143-10-200511150-00033)
10. [Roh H, Park KH, Jeon Y-J, Park SG, Lee J. Medical students’ agenda-setting abilities during medical interviews. Korean Journal of Medical Education. 2015. pp. 77–86. doi:](http://paperpile.com/b/OdchLL/K5gjH)[10.3946/kjme.2015.27.2.77](http://dx.doi.org/10.3946/kjme.2015.27.2.77)
11. [Mishra MK, Saunders CH, Rodriguez HP, Shortell SM, Fisher E, Elwyn G. How do healthcare professionals working in accountable care organisations understand patient activation and engagement? Qualitative interviews across two time points. BMJ Open. 2018;8: e023068. doi:](http://paperpile.com/b/OdchLL/Rjsql)[10.1136/bmjopen-2018-023068](http://dx.doi.org/10.1136/bmjopen-2018-023068)
12. [Coyle AC, Yen RW, Elwyn G. Interrupted opening statements in clinical encounters: A scoping review. Patient Educ Couns. 2022;105: 2653–2663. doi:](http://paperpile.com/b/OdchLL/o2yZL)[10.1016/j.pec.2022.03.026](http://dx.doi.org/10.1016/j.pec.2022.03.026)
13. [Rey-Bellet S, Dubois J, Vannotti M, Zuercher M, Faouzi M, Devaud K, et al. Agenda Setting During Follow-Up Encounters in a University Primary Care Outpatient Clinic. Health Commun. 2017;32: 714–720. doi:](http://paperpile.com/b/OdchLL/0cKyH)[10.1080/10410236.2016.1168003](http://dx.doi.org/10.1080/10410236.2016.1168003)
14. [Gobat N, Kinnersley P, Gregory JW, Pickles T, Hood K, Robling M. Measuring clinical skills in agenda-mapping (EAGL-I). Patient Educ Couns. 2015;98: 1214–1221. doi:](http://paperpile.com/b/OdchLL/on8Du)[10.1016/j.pec.2015.06.018](http://dx.doi.org/10.1016/j.pec.2015.06.018)
15. [Singh Ospina N, Phillips KA, Rodriguez-Gutierrez R, Castaneda-Guarderas A, Gionfriddo MR, Branda ME, et al. Eliciting the Patient’s Agenda- Secondary Analysis of Recorded Clinical Encounters. J Gen Intern Med. 2019;34: 36–40. doi:](http://paperpile.com/b/OdchLL/UCg3j)[10.1007/s11606-018-4540-5](http://dx.doi.org/10.1007/s11606-018-4540-5)
16. [Hoskins G, Williams B, Abhyankar P, Donnan P, Duncan E, Pinnock H, et al. Achieving Good Outcomes for Asthma Living (GOAL): mixed methods feasibility and pilot cluster randomised controlled trial of a practical intervention for eliciting, setting and achieving goals for adults with asthma. Trials. 2016;17: 584. doi:](http://paperpile.com/b/OdchLL/zmX1i)[10.1186/s13063-016-1684-7](http://dx.doi.org/10.1186/s13063-016-1684-7)
17. [Middleton JF. Asking patients to write lists: feasibility study. BMJ. 1995;311: 34. doi:](http://paperpile.com/b/OdchLL/0cx64)[10.1136/bmj.311.6996.34](http://dx.doi.org/10.1136/bmj.311.6996.34)
18. [Middleton JF, McKinley RK, Gillies CL. Effect of patient completed agenda forms and doctors’ education about the agenda on the outcome of consultations: randomised controlled trial. BMJ. 2006;332: 1238–1242. doi:](http://paperpile.com/b/OdchLL/ywJsi)[10.1136/bmj.38841.444861.7C](http://dx.doi.org/10.1136/bmj.38841.444861.7C)
19. [Mauksch LB, Hillenburg L, Robins L. The Establishing Focus protocol: Training for collaborative agenda setting and time management in the medical interview. Fam Syst Health. 2001;19: 147–157. doi:](http://paperpile.com/b/OdchLL/r4xce)[10.1037/h0089539](http://dx.doi.org/10.1037/h0089539)
20. [Brock DM, Mauksch LB, Witteborn S, Hummel J, Nagasawa P, Robins LS. Effectiveness of intensive physician training in upfront agenda setting. J Gen Intern Med. 2011;26: 1317–1323. doi:](http://paperpile.com/b/OdchLL/v3XVK)[10.1007/s11606-011-1773-y](http://dx.doi.org/10.1007/s11606-011-1773-y)
21. [Haas LJ, Glazer K, Houchins J, Terry S. Improving the effectiveness of the medical visit: a brief visit-structuring workshop changes patients’ perceptions of primary care visits. Patient Educ Couns. 2006;62: 374–378. doi:](http://paperpile.com/b/OdchLL/Rtd8N)[10.1016/j.pec.2006.06.007](http://dx.doi.org/10.1016/j.pec.2006.06.007)
22. [Stott NC, Rees M, Rollnick S, Pill RM, Hackett P. Professional responses to innovation in clinical method: diabetes care and negotiating skills. Patient Educ Couns. 1996;29: 67–73. doi:](http://paperpile.com/b/OdchLL/YBZPA)[10.1016/0738-3991(96)00935-4](http://dx.doi.org/10.1016/0738-3991(96)00935-4)
23. [Everden A, Early F, Homan K, Fuld J. P94 Patient Agenda Setting And Clinic Efficiency In Outpatients: An Individual Randomised Controlled Trial. Thorax. 2014;69: A118–A118. doi:](http://paperpile.com/b/OdchLL/uLunQ)[10.1136/thoraxjnl-2014-206260.235](http://dx.doi.org/10.1136/thoraxjnl-2014-206260.235)
24. [Higgins JPT, Thomas J, Chandler J, Cumpston M, Li T, Page MJ, et al. Cochrane Handbook for Systematic Reviews of Interventions. John Wiley & Sons; 2019. Available:](http://paperpile.com/b/OdchLL/6q958) <https://play.google.com/store/books/details?id=cTqyDwAAQBAJ>
25. [Shamseer L, Moher D, Clarke M, Ghersi D, Liberati A, Petticrew M, et al. Preferred reporting items for systematic review and meta-analysis protocols (PRISMA-P) 2015: elaboration and explanation. BMJ. 2015;350: g7647. doi:](http://paperpile.com/b/OdchLL/2vWs0)[10.1136/bmj.g7647](http://dx.doi.org/10.1136/bmj.g7647)
26. [Page MJ, McKenzie JE, Bossuyt PM, Boutron I, Hoffmann TC, Mulrow CD, et al. The PRISMA 2020 statement: an updated guideline for reporting systematic reviews. BMJ. 2021;372: n71. doi:](http://paperpile.com/b/OdchLL/peGRn)[10.1136/bmj.n71](http://dx.doi.org/10.1136/bmj.n71)
27. [Gobat NH. Agenda setting in the clinical encounter: what is it, and is it measureable? phd, Cardiff University. 2014. Available:](http://paperpile.com/b/OdchLL/LsJkr) <https://orca.cardiff.ac.uk/id/eprint/56395>
28. [Marvel MK, Epstein RM, Flowers K, Beckman HB. Soliciting the patient’s agenda: have we improved? JAMA. 1999;281: 283–287. doi:](http://paperpile.com/b/OdchLL/hmZPU)[10.1001/jama.281.3.283](http://dx.doi.org/10.1001/jama.281.3.283)
29. [Kowalski CP, McQuillan DB, Chawla N, Lyles C, Altschuler A, Uratsu CS, et al. “The Hand on the Doorknob”: Visit Agenda Setting by Complex Patients and Their Primary Care Physicians. J Am Board Fam Med. 2018;31: 29–37. doi:](http://paperpile.com/b/OdchLL/jYwZ9)[10.3122/jabfm.2018.01.170167](http://dx.doi.org/10.3122/jabfm.2018.01.170167)
30. [Ouzzani M, Hammady H, Fedorowicz Z, Elmagarmid A. Rayyan—a web and mobile app for systematic reviews. Syst Rev. 2016;5: 210. doi:](http://paperpile.com/b/OdchLL/ArbQ2)[10.1186/s13643-016-0384-4](http://dx.doi.org/10.1186/s13643-016-0384-4)
31. [Hoffmann TC, Glasziou PP, Boutron I, Milne R, Perera R, Moher D, et al. Better reporting of interventions: template for intervention description and replication (TIDieR) checklist and guide. BMJ. 2014;348: g1687. doi:](http://paperpile.com/b/OdchLL/j5RT6)[10.1136/bmj.g1687](http://dx.doi.org/10.1136/bmj.g1687)
32. [Higgins JPT, Li T, Deeks JJ. Choosing effect measures and computing estimates of effect. Cochrane Handbook for Systematic Reviews of Interventions. Wiley; 2019. pp. 143–176. doi:](http://paperpile.com/b/OdchLL/2cxgR)[10.1002/9781119536604.ch6](http://dx.doi.org/10.1002/9781119536604.ch6)
33. [Rohatgi A. WebPlotDigitizer - Copyright 2010-2022 Ankit Rohatgi. [cited 1 Jun 2023]. Available:](http://paperpile.com/b/OdchLL/6sZ0K) <https://apps.automeris.io/wpd/>
34. [Sterne JAC, Savović J, Page MJ, Elbers RG, Blencowe NS, Boutron I, et al. RoB 2: a revised tool for assessing risk of bias in randomised trials. BMJ . 2019. p. l4898. doi:](http://paperpile.com/b/OdchLL/Wbfcp)[10.1136/bmj.l4898](http://dx.doi.org/10.1136/bmj.l4898)
35. [Sterne JA, Hernán MA, Reeves BC, Savović J, Berkman ND, Viswanathan M, et al. ROBINS-I: a tool for assessing risk of bias in non-randomised studies of interventions. BMJ. 2016;355: i4919. doi:](http://paperpile.com/b/OdchLL/VvPdk)[10.1136/bmj.i4919](http://dx.doi.org/10.1136/bmj.i4919)
36. [Guyatt GH, Oxman AD, Schünemann HJ, Tugwell P, Knottnerus A. GRADE guidelines: a new series of articles in the Journal of Clinical Epidemiology. J Clin Epidemiol. 2011;64: 380–382. doi:](http://paperpile.com/b/OdchLL/Dn9eA)[10.1016/j.jclinepi.2010.09.011](http://dx.doi.org/10.1016/j.jclinepi.2010.09.011)
37. [GRADE handbook. [cited 1 Jun 2023]. Available:](http://paperpile.com/b/OdchLL/acxBM) <https://gdt.gradepro.org/app/handbook/handbook.html>
38. [Valentine JC, Pigott TD, Rothstein HR. How Many Studies Do You Need?: A Primer on Statistical Power for Meta-Analysis. J Educ Behav Stat. 2010;35: 215–247. doi:](http://paperpile.com/b/OdchLL/Em4e)[10.3102/1076998609346961](http://dx.doi.org/10.3102/1076998609346961)
39. [Chapter 10: Analysing data and undertaking meta-analyses. [cited 2 Mar 2023]. Available:](http://paperpile.com/b/OdchLL/AmJdT) <https://training.cochrane.org/handbook/current/chapter-10>
40. [Chapter 10: Analysing data and undertaking meta-analyses. [cited 31 Jul 2024]. Available:](http://paperpile.com/b/OdchLL/rxFh) <https://training.cochrane.org/handbook/archive/v6.2/chapter-10>
41. [Nikolakopoulou A, Mavridis D, Salanti G. How to interpret meta-analysis models: fixed effect and random effects meta-analyses. Evid Based Ment Health. 2014;17: 64. doi:](http://paperpile.com/b/OdchLL/FpNQ)[10.1136/eb-2014-101794](http://dx.doi.org/10.1136/eb-2014-101794)
42. [Higgins JPT, Thompson SG. Quantifying heterogeneity in a meta-analysis. Stat Med. 2002;21: 1539–1558. doi:](http://paperpile.com/b/OdchLL/gIoJW)[10.1002/sim.1186](http://dx.doi.org/10.1002/sim.1186)
43. [Chapter 13: Assessing risk of bias due to missing results in a synthesis. [cited 31 Jul 2024]. Available:](http://paperpile.com/b/OdchLL/LAfr) <https://training.cochrane.org/handbook/current/chapter-13>
44. [Popay J, Roberts H, Sowden A, Petticrew M, Arai L, Rodgers M, et al. Guidance on the conduct of narrative synthesis in systematic reviews: A product from the ESRC Methods Programme. [object Object]: Lancaster University; 2006. doi:](http://paperpile.com/b/OdchLL/MO7pT)[10.13140/2.1.1018.4643](http://dx.doi.org/10.13140/2.1.1018.4643)
45. [Campbell M, McKenzie JE, Sowden A, Katikireddi SV, Brennan SE, Ellis S, et al. Synthesis without meta-analysis (SWiM) in systematic reviews: reporting guideline. BMJ. 2020;368: l6890. doi:](http://paperpile.com/b/OdchLL/9BzOL)[10.1136/bmj.l6890](http://dx.doi.org/10.1136/bmj.l6890)
46. [Saunders CH, Sierpe A, von Plessen C, Kennedy AM, Leviton LC, Bernstein SL, et al. Practical thematic analysis: a guide for multidisciplinary health services research teams engaging in qualitative analysis. BMJ. 2023;381: e074256. doi:](http://paperpile.com/b/OdchLL/w4weq)[10.1136/bmj-2022-074256](http://dx.doi.org/10.1136/bmj-2022-074256)
