# Supplementary material for: Agenda-setting in the clinical encounter: A systematic review protocol
Source: PLoS One. 2024 Oct 24;19(10):e0312613. doi: 10.1371/journal.pone.0312613 (PMC11500969; doi:10.1371/journal.pone.0312613)
Supplement: Attachment — Submitted filename: Response to Reviewers.docx [file pone.0312613.s006.docx]

## Reviewer #1

My comments were addressed. Here is additional suggestions for improvement.

Thank you for your earlier comments, we greatly appreciate your suggestions for improvement. We have aimed to address your additional suggestions below.

1. Please discuss potential biases in the included studies, such as selection and publication bias, and outline your approach to addressing these in the review.

We appreciate your comment, and the opportunity to clarify on the issue of potential sources of bias. We have aimed to reduce selection bias as much as possible by designing an exhaustive search strategy covering both peer-reviewed and gray literature sources in collaboration with two biomedical librarians. We have highlighted this in **Secondary search strategy**, as below.

To reduce potential selection bias, we will ensure an exhaustive search by supplementing our primary search strategy with the following secondary search methods (see Supporting Information 2):

- Searching the reference lists of all included articles to find any articles not identified in our initial searches (backward citation search).
- Searching the citations of all included articles by using the ‘cited by’ feature in Web of Science (forward citation search).
- Searching Google Scholar and screening the first 25 pages of results. If results continue to be relevant after 25 pages, we will continue reviewing additional pages until two consecutive pages yield no relevant articles.
- Searching ClinicalTrials.gov for registered clinical trials.
- Searching key journals, relevant academic conference proceedings, and gray literature such as technical reports and works in progress.
- Searching reference lists provided by colleagues with expertise in research on clinical visit agenda-setting.

We discuss our approach to assessing reporting / publication bias in **Data synthesis**, as below.

For outcomes reported by at least 10 studies, we will assess reporting bias using funnel plots, where effect estimates of the common outcome measure are plotted against trial sample size. We will then conduct Egger’s regressions to statistically assess for asymmetry, assuming significance at p<0.05. For outcomes reported by fewer than 10 studies, we will assess reporting bias qualitatively.[[1]](https://paperpile.com/c/vTekCZ/4Bddo)

2. Additionally, clarify how you will evaluate gray literature alongside peer-reviewed evidence, including your assessment of their reliability. It would be beneficial to express your confidence even in peer-reviewed studies and how you plan to integrate both types of literature in your analysis.

Thank you for your suggestion. We have clarified that both peer-reviewed articles and gray literature will be evaluated for their methodological quality using the same approach in **Methodological quality assessment**, as below.

As our inclusion criteria cover multiple study designs, we will use two different risk-of-bias tools to evaluate the included studies: RoB 2 (the Cochrane risk-of-bias tool) for randomized control trials,[[2]](https://paperpile.com/c/vTekCZ/qvH0U) and ROBINS-I (Risk Of Bias In Non-randomised Studies - of Interventions) for non-randomized trials.[[3]](https://paperpile.com/c/vTekCZ/OVncM) All reviewers will be trained on using the tools before beginning their assessments. Two independent reviewers will assign each study a rating of methodological quality using the risk-of-bias tool corresponding to its design. A third independent reviewer will resolve any disagreements. We will also use GRADE (Grading of Recommendations, Assessment, Development and Evaluation) to evaluate the certainty of the evidence reported.[[4,5]](https://paperpile.com/c/vTekCZ/FiWJj+Lngoo) We will use the RoB 2 and ROBINS-I results and other components of the included studies to apply the GRADE scoring. We will use this same approach to assessing methodological quality for both peer-reviewed articles and gray literature.

We have also specified that all studies, including those from both peer-reviewed articles and gray literature, will be integrated into the analysis in the same way. Please see **Data synthesis**, as below.

All included studies, regardless of their source, will be synthesized using the same approach. For any outcomes reported by at least two studies, we will pool the results and conduct a meta-analysis using statistical software.[[6,7]](https://paperpile.com/c/vTekCZ/wPfGx+JXrkW) We will present dichotomous data as relative risks and continuous data as means or standardized mean differences, with all analyses using 95% confidence intervals. As it is unlikely that the intervention effects will be identical, we will use a random effects model, assuming significance at p<0.05.[[7,8]](https://paperpile.com/c/vTekCZ/aQJtY+JXrkW)

3. I suggest elaborating on the specific qualitative synthesis methods you plan to employ. Providing details on how you will analyze and interpret the qualitative findings, as well as how you will ensure rigor and credibility, will strengthen the protocol.

Thank you for your comment. We have discussed our approach to qualitatively synthesizing the data in **Data synthesis**, as below. To ensure transparency and rigor, we will use widely recommended reporting criteria, Synthesis Without Meta-analysis.[[9]](https://paperpile.com/c/vTekCZ/Q9lai) We will also engage with research partners who were not otherwise involved in the systematic review to take part in the qualitative synthesis process.

We will also produce a narrative synthesis of the included studies.[[10]](https://paperpile.com/c/vTekCZ/f9j3l) The data from each study, including intervention characteristics, setting, participant characteristics, and enrollment metrics, will be used to build evidence tables according to the SWiM (Synthesis Without Meta-analysis) reporting guidelines.[[9]](https://paperpile.com/c/vTekCZ/Q9lai) Using the evidence tables, we will qualitatively summarize the direction and intensity of the effects of the agenda-setting interventions. We will also summarize results relating to our secondary aims. To increase the validity of our narrative synthesis, we will adopt the participatory approach outlined in Practical Thematic Analysis[[11]](https://paperpile.com/c/vTekCZ/lsGmb) and will include a clinician and a patient partner as members of the research team. Our partners will take part in interpreting and summarizing the results of the included studies, as well as provide feedback on the initial results of the systematic review.

## Reviewer #2

The research questions to be addressed is clear and able to add value to the knowledge base. The effectiveness of agenda-setting interventions in improving healthcare outcomes is not clear and I couldn't get a systematic review that has examined clinical visit agenda-setting interventions. The protocol is technically and methodologically sound to conduct the stated systematic review. The the search, study selection and extraction methods describes ensures the feasibility to replicate. So, I suggest accepting this protocol to be published.

We sincerely appreciate your earlier suggestions on improving our protocol, and thank you for your comments. We hope that the systematic review will contribute to the literature on the effectiveness of these agenda-setting interventions.

## References

1.	[Chapter 13: Assessing risk of bias due to missing results in a synthesis. [cited 31 Jul 2024]. Available:](http://paperpile.com/b/vTekCZ/4Bddo) <https://training.cochrane.org/handbook/current/chapter-13>

2.	[Sterne JAC, Savović J, Page MJ, Elbers RG, Blencowe NS, Boutron I, et al. RoB 2: a revised tool for assessing risk of bias in randomised trials. BMJ. 2019;366: l4898. doi:](http://paperpile.com/b/vTekCZ/qvH0U)[10.1136/bmj.l4898](http://dx.doi.org/10.1136/bmj.l4898)

3.	[Sterne JA, Hernán MA, Reeves BC, Savović J, Berkman ND, Viswanathan M, et al. ROBINS-I: a tool for assessing risk of bias in non-randomised studies of interventions. BMJ. 2016;355: i4919. doi:](http://paperpile.com/b/vTekCZ/OVncM)[10.1136/bmj.i4919](http://dx.doi.org/10.1136/bmj.i4919)

4.	[Guyatt GH, Oxman AD, Schünemann HJ, Tugwell P, Knottnerus A. GRADE guidelines: a new series of articles in the Journal of Clinical Epidemiology. J Clin Epidemiol. 2011;64: 380–382. doi:](http://paperpile.com/b/vTekCZ/FiWJj)[10.1016/j.jclinepi.2010.09.011](http://dx.doi.org/10.1016/j.jclinepi.2010.09.011)

5.	[GRADE handbook. [cited 1 Jun 2023]. Available:](http://paperpile.com/b/vTekCZ/Lngoo) <https://gdt.gradepro.org/app/handbook/handbook.html>

6.	[Valentine JC, Pigott TD, Rothstein HR. How Many Studies Do You Need?: A Primer on Statistical Power for Meta-Analysis. J Educ Behav Stat. 2010;35: 215–247. doi:](http://paperpile.com/b/vTekCZ/wPfGx)[10.3102/1076998609346961](http://dx.doi.org/10.3102/1076998609346961)

7.	[Chapter 10: Analysing data and undertaking meta-analyses. [cited 2 Mar 2023]. Available:](http://paperpile.com/b/vTekCZ/JXrkW) <https://training.cochrane.org/handbook/current/chapter-10>

8.	[Nikolakopoulou A, Mavridis D, Salanti G. How to interpret meta-analysis models: fixed effect and random effects meta-analyses. Evid Based Ment Health. 2014;17: 64. doi:](http://paperpile.com/b/vTekCZ/aQJtY)[10.1136/eb-2014-101794](http://dx.doi.org/10.1136/eb-2014-101794)

9.	[Campbell M, McKenzie JE, Sowden A, Katikireddi SV, Brennan SE, Ellis S, et al. Synthesis without meta-analysis (SWiM) in systematic reviews: reporting guideline. BMJ. 2020;368: l6890. doi:](http://paperpile.com/b/vTekCZ/Q9lai)[10.1136/bmj.l6890](http://dx.doi.org/10.1136/bmj.l6890)

10.	[Popay J, Roberts H, Sowden A, Petticrew M, Arai L, Rodgers M, et al. Guidance on the conduct of narrative synthesis in systematic reviews: A product from the ESRC Methods Programme. [object Object]: Lancaster University; 2006. doi:](http://paperpile.com/b/vTekCZ/f9j3l)[10.13140/2.1.1018.4643](http://dx.doi.org/10.13140/2.1.1018.4643)

11.	[Saunders CH, Sierpe A, von Plessen C, Kennedy AM, Leviton LC, Bernstein SL, et al. Practical thematic analysis: a guide for multidisciplinary health services research teams engaging in qualitative analysis. BMJ. 2023;381: e074256. doi:](http://paperpile.com/b/vTekCZ/lsGmb)[10.1136/bmj-2022-074256](http://dx.doi.org/10.1136/bmj-2022-074256)
